# Seasonal ecological quality in China: concurrent and lagged drought responses and dominant factors

**DOI:** 10.3389/fpls.2026.1850459

**Published:** 2026-08-24

**Authors:** Wenhan Pei, Jiping Liu, Wanhe Lu, Han Guo, Yu Wang, Yanhui Chen

**Affiliations:** 1 College of Geographic Science and Tourism, Jilin Normal University, Siping, China; 2 Jilin Provincial Key Laboratory of Eco-Environmental Remote Sensing Big Data, Changchun, China

**Keywords:** ecology quality, remote sensing ecological index (RSEI), seasonal drought, standardized precipitation evapotranspiration index (SPEI), lagged response, dry-wet regions, seasonal difference

## Abstract

**Introduction:**

Global warming is intensifying seasonal droughts and increasing their pressure on terrestrial ecosystems. However, most assessments of drought impacts on ecological quality remain focused on annual-scale or concurrent relationships, with insufficient attention to drought seasonality, lagged effects, and differences along aridity-humidity gradients.

**Methods:**

This study constructed spring, summer, and autumn Remote Sensing Ecological Index (RSEI) for China from 2001 to 2022, and analyzed the spatiotemporal variation characteristics of ecological quality at the seasonal scale. Meanwhile, by integrating the Standardized Precipitation Evapotranspiration Index (SPEI), other climatic variables, and human activity data, we investigated the concurrent and lagged effects of seasonal drought on ecological quality, incorporated these effects into the subsequent driving-factor analysis of ecological quality changes, and further compared drought responses and dominant-factor patterns across different dry-wet regions.

**Results and discussion:**

The mean RSEI in spring, summer, and autumn all increased significantly, with rates of 0.0032, 0.0017, and 0.0012 yr^-1^, respectively (p < 0.01). The significant response area of seasonal RSEI to SPEI was largest in summer, accounting for 30.15% of the study area, followed by spring and autumn, accounting for 26.78% and 23.53%, respectively. Spring RSEI was mainly affected by concurrent spring SPEI, summer RSEI was mainly affected by antecedent spring drought, and autumn RSEI was mainly affected by antecedent winter and summer droughts. Drought responses differed markedly among dry-wet regions. In spring, the significant impact range of drought on ecological quality generally decreased with increasing aridity, whereas in summer it was highest in semi-arid regions. In autumn, RSEI and SPEI were mainly negatively correlated in humid and semi-humid regions, but mainly positively correlated in semi-arid and arid regions. After accounting for lagged drought effects, SPEI was the dominant factor with the widest significant influence range across all three seasons, while temperature, human footprint, and soil moisture were the main secondary factors in spring, summer, and autumn, respectively. Seasonal ecological quality monitoring, drought-risk identification, and regional ecological conservation should therefore consider the season of drought occurrence, antecedent water conditions, and regional dry-wet background. These findings can provide scientific support for seasonal ecological monitoring, regionalized drought-risk management, and differentiated ecological conservation strategies in China.

## 1 Introduction

Ecological quality serves as a crucial indicator for assessing regional ecological conditions and sustainable development levels, reflecting the suitability of ecosystems for human survival and the sustainable development of the social economy within a specific spatiotemporal range (Strassburg et al., 2020; Zhen et al., 2025). Changes in ecological quality are driven by multiple factors including climate, topography, and human activities (Yuan et al., 2021; Bai et al., 2023). Climate factors not only serve as fundamental conditions for the formation and evolution of ecological environments (Zhang et al., 2023), but also act as the dominant influence on ecological quality in certain regions (Wang et al., 2022; Kang et al., 2024; Tang et al., 2024b). Rising temperatures, abnormal precipitation patterns, and frequent extreme weather events can significantly impact the structure, function, provisioning, and regulating services of ecosystems, further exacerbating the vulnerability of the ecological environment (Schwalm et al., 2017; Wu et al., 2025a). In addition, recent studies have shown that the long-term impacts of climatic factors on ecosystems are expected to be several times higher than currently observed levels (Lee et al., 2024). Therefore, it is of great theoretical and practical significance for ecological conservation to explore the influence of climatic factors on ecological quality.

Drought, one of the most pervasive and destructive climate factors (Hao et al., 2018), has been shown to be a key driver of ecological quality (Chen et al., 2023; Li et al., 2023b; Zhang et al., 2024). Drought can adversely affect ecological quality by weakening water supplies, exacerbating soil erosion and land degradation, inducing wildfires and leading to forest die-offs, and inhibiting vegetation growth, reducing net primary productivity and carbon storage capacity, among other things (Vicente-Serrano et al., 2020; Rahman et al., 2025). With global warming, the frequency and intensity of droughts will continue to increase in the future, and they may pose an even greater threat to ecosystems (Chen et al., 2025; Xiao et al., 2025). However, most of the current studies focus on the response of ecological quality to annual-scale droughts, while neglecting the importance and extent of seasonal droughts on ecological quality (Li et al., 2023b, 2024). It has been shown that seasonal droughts can have significant impacts on ecosystems (Bastos et al., 2020; Niu and Liu, 2021; Hao et al., 2024). For example, Deng et al. (2023) found that summer drought led to a significant decrease in leaf area index (LAI), gross primary productivity (GPP) and net ecosystem productivity (NEP). In particular, the increasing frequency and intensity of seasonal droughts globally implies that the impacts of seasonal droughts on ecological quality may be further exacerbated (Senf et al., 2020; Li et al., 2021b). Therefore, there is an urgent need for a comprehensive understanding of the impacts of seasonal droughts on ecological quality.

Due to differences in regional ecohydrological conditions, climate, and ecosystem drought tolerance, the impacts of drought on ecosystems show clear seasonal differences (Huang et al., 2018; Huang and Zhai, 2024). For example, Wu et al. (2022) observed that summer and autumn droughts had stronger negative effects on vegetation productivity in China from 2001 to 2020. Meanwhile, drought impacts also exhibit marked differences along dry-wet gradients. Jiao et al. (2021) pointed out that drought limitation on vegetation growth mainly occurs in water-deficit regions, whereas water is not necessarily the primary limiting factor in humid regions. Using PDSI, SPEI, and NDVI data from 293 natural vegetation sites in China, Zhang and Zhang (2019) further showed that drought vulnerability of natural vegetation increases with aridity but decreases again in extremely arid regions. In addition, the impacts of drought on ecosystems are often not immediate, but may exhibit lagged effects through soil moisture storage, root-zone water availability, and vegetation recovery processes (Wu et al., 2015). Such seasonal differences and dry-wet gradient differences in drought impacts may be further reflected in changes in ecological quality. Therefore, studies based only on annual-scale drought or concurrent seasonal drought are insufficient for fully identifying the responses of ecological quality to seasonal drought across different dry-wet regions, thereby limiting the effectiveness of ecological conservation and drought risk management measures.

The Remote Sensing Ecological Index (RSEI) integrates indices of greenness, heat, dryness, and humidity extracted directly from satellite imagery (Xu, 2013). It avoids biases in weighting definitions caused by individual characteristics while accounting for ecosystem integrity, complexity, and diversity, and is commonly used for regional ecological quality assessments (Yang et al., 2021; An et al., 2022; Geng et al., 2022; Yi et al., 2023). In recent years, approaches combining RSEI with drought indices have been increasingly used to assess the response of ecological quality to drought. For example, Yan et al. (2024) analyzed the relationship between RSEI and SPEI and found that ecological quality in the southeastern, southwestern, and northern Qinghai-Tibet Plateau was relatively sensitive to meteorological drought. Li et al. (2023b) analyzed the effects of carbon-water cycles and human activities on RSEI changes and identified drought as one of the key driving factors of RSEI. Qin et al. (2024) quantified the contribution of drought to RSEI changes in the Yellow River Basin and reported that EEQ was mainly positively affected by spring, autumn, and winter droughts, but negatively affected by summer drought. These studies have deeply explored the major impacts of drought on ecological quality. However, most existing RSEI-based assessments still focus on annual ecological quality or concurrent drought relationships. This overlooks the possibility that seasonal RSEI may respond differently to spring, summer, and autumn droughts, and that antecedent drought conditions may continue to affect ecological quality after the season in which drought occurs. Therefore, a seasonal and lag-response perspective that incorporates dry-wet regional differences is needed to more accurately assess the impacts of drought on ecological quality.

China has the third largest land area in the world, spanning a broad range of latitudes and encompassing varied landforms such as mountains and plateaus, along with multiple climate zones (Ding et al., 2024). In recent decades, due to the increasing frequency of seasonal droughts (Ma et al., 2015; Xu et al., 2021), the ecological environment in China has been seriously and adversely affected (Deng et al., 2023). For instance, the autumn, winter, and spring droughts in southwest China from 2009 to 2010 suppressed vegetation growth, leading to a significant reduction in vegetation productivity (GPP, 5.7 ± 9.5 gCm^-2^month^-1^) and carbon uptake (4.4 ± 5 gCm^-2^ month^-1^) (Li et al., 2019). The severe seasonal drought in 2022 resulted in reduced crop yields across more than 600,000 hectares in China’s Yangtze River Basin (Hao et al., 2024). Therefore, China provides a suitable national-scale setting for examining how ecological quality responds to seasonal drought, how such responses are affected by lagged drought conditions, and how they vary across aridity zones.

To address the above gaps, this study constructed spring, summer, and autumn RSEI datasets for China from 2001 to 2022 and combined them with seasonal SPEI and multiple climatic and anthropogenic factors to systematically assess the relationship between seasonal drought and ecological quality changes. Specifically, this study aimed to: (1) reveal the spatiotemporal variation characteristics of seasonal RSEI and SPEI in China; (2) explore the concurrent and lagged effects of seasonal SPEI on RSEI and their spatial heterogeneity; and (3) evaluate the dominant factors of seasonal RSEI, including drought and other natural and anthropogenic factors, based on the incorporation of lagged SPEI responses, and further analyze their differences across dry-wet regions. This study can help clarify how seasonal ecological quality responds to drought across different dry-wet regions and provide evidence for ecological conservation and drought risk management in different regions.

## 2 Materials and methods

### 2.1 Study area overview

**Figure 1:**
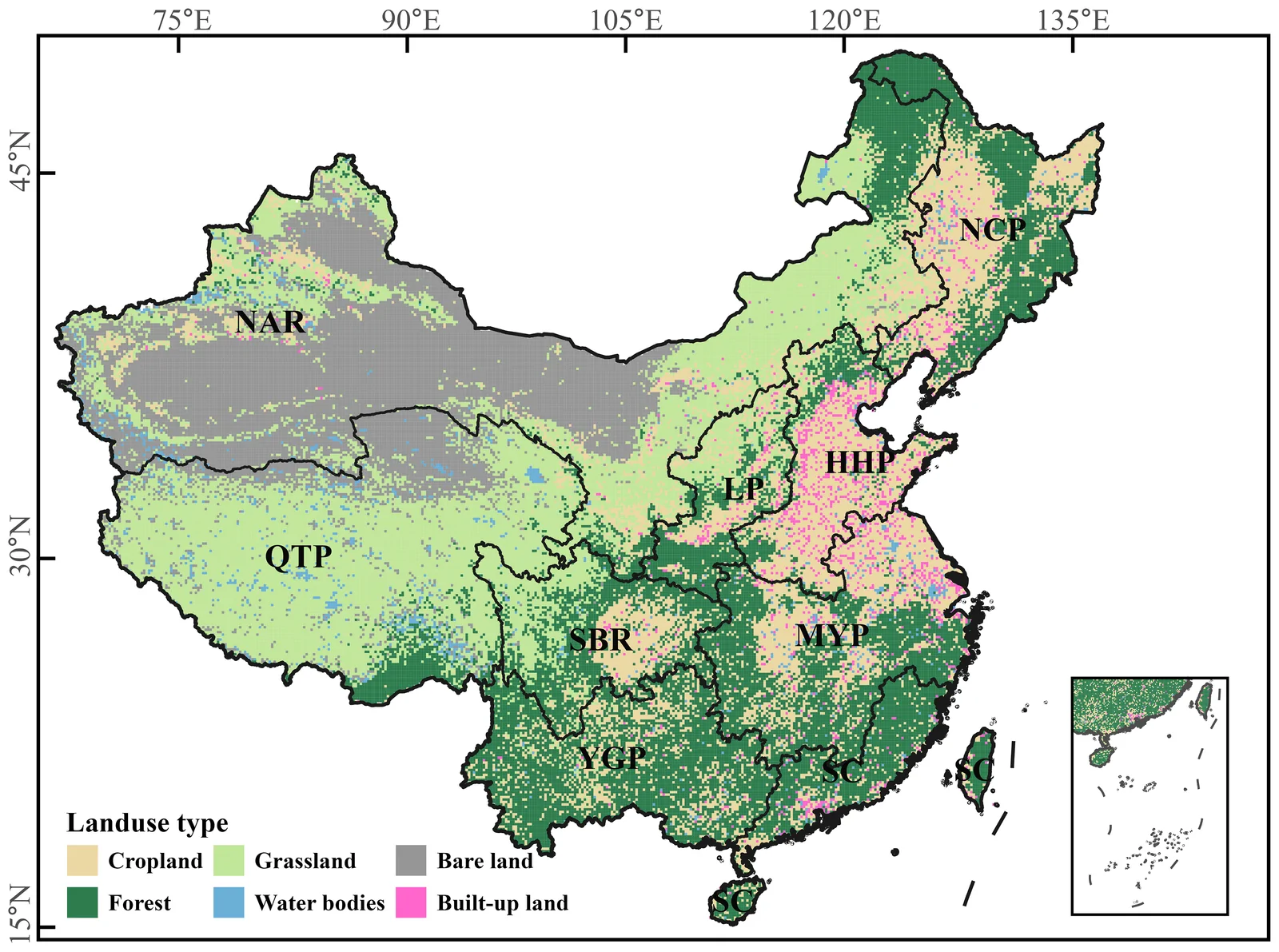
Nine geographical regions and land cover (2022) across China.

China has a vast territory, located in the southeast of Eurasia, covering an area of about 9.6 million square kilometers (Wang et al., 2023a). It is deeply affected by warm and humid air currents in the Pacific Ocean and Indian Ocean. It has an obvious monsoon climate, presenting a climate change from warm-wet to cold-dry from southeast to northwest, with four distinct seasons (**Figure 1**) (Li et al., 2023b). China’s terrain is characterized by high in the west and low in the east (Xin et al., 2024). Significant spatial variations in temperature, precipitation, and topography lead to pronounced heterogeneity in land cover types across China (Wu et al., 2022), mainly grassland and forest (**Figure 1**), accounting for more than 55% of the total area (Dilixiati et al., 2025). The vegetation cover is low in the northwest and high in the southeast (Liu et al., 2021).

Drought characteristics differ markedly among climatic regions in China. In the arid region of northwestern China, precipitation is scarce, solar radiation is strong, atmospheric evaporative demand is high, and water resources are relatively limited. Droughts in this region are usually characterized by long duration and wide spatial extent, and their formation is mainly associated with persistent precipitation deficits and strong evapotranspiration (Li et al., 2026). The semi-arid region of northern China is located between the arid inland region of northwestern China and the humid and semi-humid monsoon regions of eastern China, forming an important climatic transition zone. This region receives relatively low precipitation, with large interannual variability, and precipitation is mainly concentrated in the warm season. Even short-term precipitation deficits can rapidly reduce regional soil moisture and further develop into agricultural or ecological drought (Li et al., 2021a). In the eastern and southern monsoon regions of China, annual precipitation is generally abundant, but its seasonal and interannual distribution is highly uneven (Xia et al., 2017). Drought occurrence in this region is closely related to anomalies in the onset timing, intensity, duration, and withdrawal of the monsoon, and is also affected by changes in the spatial distribution of monsoon precipitation (Qian et al., 2009). Northern and northeastern China are more prone to spring drought or early-summer drought, while the Huang-Huai-Hai Plain is vulnerable to seasonal water deficits during key crop growth stages (Liu et al., 2010). In the middle and lower reaches of the Yangtze River and South China, summer or autumn drought may occur when the subtropical high persists and precipitation is suppressed (Cai et al., 2022). The Qinghai-Tibet Plateau has unique drought formation and evolution characteristics because of its high elevation, low temperature, strong solar radiation, complex terrain, and widespread cryospheric development (Wu et al., 2025b). Droughts on the Plateau are influenced not only by precipitation variability and atmospheric evaporative demand, but also closely related to changes in snow cover, glacier meltwater, permafrost conditions, and seasonal freeze-thaw processes (Sun et al., 2026).

According to climatic and physical geographical characteristics, the study area was divided into nine subregions. **Figure 1** shows nine geographical regions of China divided according to regional differences, namely Northeast China Plain (NCP), Northern arid and semiarid region (NAR), Huang-Huai-Hai Plain (HHP), Loess Plateau (LP), Qinghai Tibet Plateau (QTP), Middle-lower Yangtze Plain (MYP), Sichuan Basin region (SBR), Yunnan-Guizhou Plateau (YGP), and Southern China (SC) (Wang et al., 2023a).

### 2.2 Data sources

#### 2.2.1 RSEI

The Remote Sensing Ecological Index (RSEI) is employed to comprehensively evaluate the ecological quality of the study area. We obtained the data needed to build RSEI from the GEE platform (https://earthengine.google.com/). Google Earth Engine (GEE) is a cloud-based geospatial platform that supports large-scale data analysis and provides access to extensive open datasets, reducing the need for local data downloads and storage. We employed the computational methods proposed by Xu (2013), selecting four indices that can collectively describe regional ecological quality (greenness, heat, dryness, and humidity), and performed cloud removal, cropping, projection, and calculation on them. Furthermore, to mitigate the effect of extensive water bodies on normalized and index eigenvalues, a modified normalized water index (MNDWI) was used to mask water body regions prior to calculation (Xu, 2007). To ensure temporal consistency with the seasonal drought analysis, spring, summer, and autumn RSEI were reconstructed separately for each year from 2001 to 2022 using observations from March–May, June–August, and September–November, respectively. The specific datasets used are summarized in **Table 1**, and detailed procedures for quality control, seasonal compositing, water masking, indicator calculation, normalization, and principal component analysis are provided in the **Supplementary Material**.

**Table 1:** The specific data sources and corresponding satellite products for each indicator.

Indicator	Representation	Satellite product	Spatial resolution	Temporal resolution	Data period
Greenness	NDVI (Normalized Difference Vegetation Index)	MOD13A1	500 m	16-day	2001–2022
Heat	LST (Land Surface Temperature)	MOD11A2 (LST_DAY_1km)	1 km	8-day	2001–2022
Dryness	NDBSI (Normalized Difference Building and Soil Index)	MOD09A1	500 m	8-day	2001–2022
Humidity	WET	MOD09A1	500 m	8-day	2001–2022
Water Mask	MNDWI (Modified Normalized Difference Water Index)	MOD09A1 (‘sur_refl_b04’, ‘sur_refl_b06’)	500 m	8-day	2001–2022

#### 2.2.2 SPEI

Drought index is one of the most commonly used methods for drought monitoring and assessment. In this study, the Standardized Precipitation Evapotranspiration Index (SPEI) was employed to characterize drought conditions. SPEI has demonstrated high effectiveness in drought monitoring and has been widely applied in numerous studies (Gumus, 2023; Li et al., 2024c; Wang et al., 2025). The SPEI data for 2001–2022 were provided by Zhang et al. (2025) with 0.1 degree spatial resolution and monthly time resolution, which shows high consistency with drought indices calculated from Climatic Research Unit (CRU) and China Meteorological Forcing Dataset (CN05.1) data.

This study selected SPEI as the main indicator of seasonal drought, mainly because SPEI can simultaneously account for water supply and atmospheric water demand rather than reflecting only precipitation anomalies. Compared with drought indices based solely on precipitation, SPEI characterizes drought intensity through the water balance between precipitation and potential evapotranspiration, making it more suitable for identifying ecosystem water stress under climate warming (Wang et al., 2018; Vicente-Serrano et al., 2020). In addition, RSEI is composed of remote sensing indicators of greenness, wetness, heat, and dryness, and can reflect the integrated response of ecological quality to water availability, land-surface hydrothermal conditions, and vegetation growth status (Xu, 2013). Therefore, using SPEI, which is based on the imbalance between atmospheric water supply and demand, helps explain changes in seasonal RSEI from the perspective of climatic water stress. SPEI also has multi-timescale characteristics and is suitable for identifying seasonal differences and lagged responses in the impacts of meteorological drought on ecosystems, which is consistent with the objective of this study to analyze seasonal drought and its antecedent effects (Gumus, 2023; Wang et al., 2023b; Wei et al., 2023).

Seasonal drought was quantified using the 3-month Standardized Precipitation Evapotranspiration Index (SPEI-3). Specifically, SPEI-3 values in May, August, November, and February (of the subsequent year) were taken to represent drought conditions in spring (March–May), summer (June–August), autumn (September–November), and winter (December–February), respectively.

#### 2.2.3 Land use data

Land-use data for 2001 and 2022 were obtained from the China Land Cover Dataset (CLCD), an annual land-cover product developed by Yang and Huang (2021) based on 335,709 Landsat scenes processed on Google Earth Engine (GEE). The CLCD has a spatial resolution of 30 m with an overall accuracy of 79.31%. All data were resampled to a uniform resolution of 0.1 degree using bilinear interpolation and nearest neighbor resampling for land use data.

#### 2.2.4 Other climatic and human activity data

The Aridity Index (AI) characterizes the long-term balance between regional water supply and atmospheric water demand and is an important indicator for identifying dry-wet climatic backgrounds. In this study, we used the annual Aridity Index dataset for China, with a spatial resolution of 0.0083333°, obtained from the National Tibetan Plateau Data Center (https://doi.org/10.11888/Atmos.tpdc.300560) (Peng et al., 2017). The dataset was calculated as the ratio of annual potential evapotranspiration to annual precipitation, namely AI = PET/PRE, with larger values indicating drier conditions. It was used to identify arid, semi-arid, semi-humid, and humid regions. We used annual AI data from 2001 to 2022, resampled them to a spatial resolution of 0.1° using bilinear interpolation, calculated the multi-year mean AI, and classified the study area into humid (AI < 1), semi-humid (1 ≤ AI < 1.5), semi-arid (1.5 ≤ AI < 4), and arid (AI ≥ 4) regions according to the AI thresholds (**Supplementary Figure 1**) (Peng et al., 2017).

Human Footprint reflects the comprehensive pressure exerted by human activities on ecological processes and natural landscapes. The dataset was developed by Mu et al. (2022) using eight indicators representing different dimensions of human pressure, including built-up areas, population density, nighttime lights, cropland, pasture, roads, railways, and navigable waterways, producing a global annual Human Footprint dataset at a spatial resolution of 1 km. This dataset is highly consistent with existing historical Human Footprint products and provides important basic data support for studies related to human activities and changes in natural resources. Because seasonal-scale human activity intensity data for China are difficult to obtain, we used the annual Human Footprint data from 2001 to 2022 as an indicator of human activity intensity and assigned the same annual Human Footprint value to the spring, summer, and autumn RSEI analyses of the corresponding year.

Monthly precipitation, air temperature, and surface downward shortwave radiation data at a spatial resolution of 0.1° were obtained from the European Centre for Medium-Range Weather Forecasts Reanalysis v5-Land (ERA5-Land) dataset (Muñoz-Sabater et al., 2021). ERA5-Land is a high-resolution land-surface climate dataset generated by the European Centre for Medium-Range Weather Forecasts (ECMWF) through a land surface model based on the ERA5 reanalysis. It provides long-term, spatially continuous, and physically consistent land-surface hydrothermal and radiation variables and has been widely used in climate, hydrology, agriculture, and ecological-environmental studies (Forzieri et al., 2022; Li et al., 2024c; Xu et al., 2024). Soil moisture was obtained from GLEAM v4.3a. GLEAM4 is the fourth-generation global land evaporation and soil moisture dataset generated by the Global Land Evaporation Amsterdam Model, providing monthly soil moisture data at a spatial resolution of 0.1° since 1980 (Miralles et al., 2025). In this study, monthly precipitation, air temperature, radiation, and soil moisture data were composited into spring, summer, and autumn mean values to match the RSEI and SPEI datasets.

### 2.3 Methods

#### 2.3.1 Calculate remote sensing ecological index

First, four indicators (NDVI, WET, LST, and NDBSI) constituting the RSEI were calculated. To eliminate dimensional differences between the indicators, all four were normalized. Principal Component Analysis (PCA) was then applied to obtain the first principal component value, which was subsequently normalized to derive the final RSEI. The overall construction of RSEI from NDVI, WET, LST, and NDBSI is summarized in **Equation 1**, following (Xu, 2013):

(1) RSEI=f(NDVI,WET,LST,NDBSI)

RSEI values range between 0 and 1, with higher RSEI indicating higher ecology quality. The detailed computation of each indicator and the normalization process are provided in the **Supplementary Material**.

#### 2.3.2 The Mann-Kendall test and Sen’s slope estimation

The Mann-Kendall test method, combined with Sen’s slope estimation method, provides a powerful statistical analysis of trends in remote sensing data time series (Li et al., 2014; Huang et al., 2022).The Mann-Kendall test was used to assess the significance of trend analysis results (Mann, 1945; Kendall, 1975; Hirsch et al., 1982). Sen’s slope estimation method was used to quantitatively assess the spatiotemporal trends of RSEI and SPEI in the study area from 2001 to 2022 (Sen, 1968). The specific calculation formula is set out in **Supplementary Material**. The criteria for evaluating RSEI and SPEI trends in this study are detailed in **Table 2**.

**Table 2:** Classification of RSEI and SPEI trends from 2001 to 2022.

β	Z range	Trend type	Trend features
β > 0	Z > 1.96	SI	Significant increase
	Z ≤ 1.96	NSI	Non-significant increase
β < 0	Z ≤ -1.96	SD	Significant decrease
	Z > -1.96	NSD	Non-significant decrease

When |Z| ≥ 1.96, the result passes the 95% confidence level test.

#### 2.3.3 Lagged partial correlation analysis

In this study, we analyzed the relationships between spring, summer, and autumn RSEI and concurrent and antecedent seasonal SPEI-3 to investigate the lagged response of seasonal ecological quality to drought. Because seasonal RSEI is simultaneously affected by multiple climatic and human activity factors, partial correlation analysis was used to calculate the correlation between RSEI and SPEI-3 while excluding the interference of other variables (Baba et al., 2004). The partial correlation coefficient was calculated using **Equation 2**:

(2) $$R_{xy,z}=\frac{R_{xy}-R_{xz}R_{yz}}{\sqrt{1-R_{xz}^{2}}\sqrt{1-R_{yz}^{2}}}$$

where *x*, *y*, and *z* represent seasonal RSEI, seasonal drought, and the control variables, respectively; *R_xy,z_* represents the partial correlation coefficient between *x* and *y* after controlling for *z*; and *R_xy_*, *R_xz_*, and *R_yz_* represent the correlation coefficients between the corresponding variables. The control variables included air temperature, precipitation, solar radiation, soil moisture, and human activities.

For different target seasons, concurrent and antecedent seasonal SPEI-3 series were set separately. Spring RSEI was subjected to partial correlation analysis with SPEI-3 in the previous summer, previous autumn, winter, and concurrent spring; summer RSEI was subjected to partial correlation analysis with SPEI-3 in the previous autumn, winter, spring, and concurrent summer; and autumn RSEI was subjected to partial correlation analysis with SPEI-3 in winter, spring, summer, and concurrent autumn. The partial correlation coefficients between RSEI and SPEI-3 in different candidate seasons were calculated pixel by pixel. When the partial correlation relationship reached the significance level (p < 0.05), the season corresponding to the partial correlation coefficient with the largest absolute value among the candidate seasons was identified as the dominant lag season of seasonal RSEI response to SPEI-3, and the positive or negative direction of this partial correlation coefficient was retained.

#### 2.3.4 Multifactor partial correlation analysis

To identify the dominant factors of seasonal RSEI changes after considering lagged drought responses, this study further conducted pixel-wise multifactor partial correlation analysis. The factors included in the analysis were drought in the dominant lag season determined in Section 2.3.3, air temperature, precipitation, solar radiation, soil moisture, and human activities. To examine potential collinearity among these factors, the variance inflation factor (VIF) was used before the partial correlation analysis. The VIF values of all variables were lower than 3, far below the threshold of 5, indicating no obvious collinearity among the variables and supporting their use in the subsequent multifactor partial correlation analysis (Tian and Tian, 2026).

For each pixel, the partial correlation coefficients between seasonal RSEI and each of the above factors were calculated separately. When calculating the partial correlation between RSEI and a given factor, the remaining factors were used as control variables. For example, when calculating the partial correlation coefficient between RSEI and drought in the dominant lag season, air temperature, precipitation, solar radiation, soil moisture, and human activities were controlled. When calculating the partial correlation coefficient between RSEI and air temperature, drought in the dominant lag season and the remaining climatic and human activity factors were controlled. When the partial correlation relationship reached the significance level (p < 0.05), the factor with the largest absolute partial correlation coefficient was identified as the dominant factor of seasonal RSEI changes for that pixel.

## 3 Results

### 3.1 Spatial and temporal variation characteristics of seasonal RSEI and SPEI

The principal component analysis (PCA) results for the spring, summer, and autumn RSEI from 2001 to 2022 showed that the first principal component (PC1) explained 61.90%–85.16% of the total variance, with an overall mean contribution rate of 72.95%. Among the three seasons, summer had the highest mean PC1 contribution rate (83.16%), followed by spring (68.53%) and autumn (67.18%) (**Supplementary Tables 1**–**S3**). The signs of the PC1 loadings were generally consistent with the expected ecological effects of the four indicators. NDVI and WET exhibited positive loadings in all seasons, with mean values of 0.909 and 0.356, respectively, indicating that vegetation greenness and surface moisture contributed positively to ecological quality. In contrast, LST and NDBSI generally exhibited negative loadings, with mean values of −0.172 and −0.016, respectively, reflecting the adverse effects of surface heat and dryness on ecological conditions (**Supplementary Tables 1**–**S3**). These results demonstrate that PC1 effectively integrated the ecological information contained in the four indicators and that the resulting seasonal RSEI reasonably represented the ecological environmental quality of the study area.

**Figure 2:**
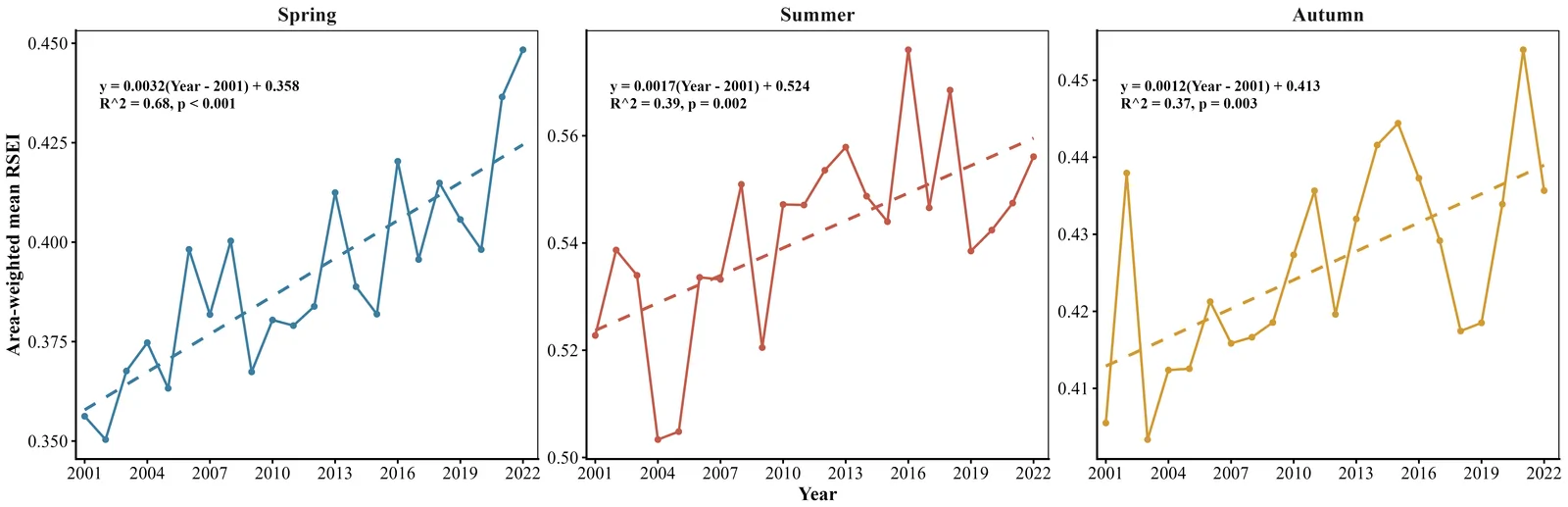
Temporal trends of mean seasonal RSEI in China from 2001 to 2022.

To characterize the temporal evolution of seasonal ecological quality at the national scale, the mean RSEI values for spring, summer, and autumn were calculated for each year from 2001 to 2022, and their temporal trends were estimated using linear regression (**Figure 2**). The national mean RSEI increased significantly in all three seasons (p < 0.01), indicating an overall improvement in China’s seasonal ecological quality during the study period. The largest increase occurred in spring, with a trend of 0.0032 yr^-1^ (R² = 0.68, p < 0.01). This was followed by summer and autumn, with trends of 0.0017 yr^-1^ (R² = 0.39, p < 0.01) and 0.0012 yr^-1^ (R² = 0.37, p < 0.01), respectively.

**Figure 3:**
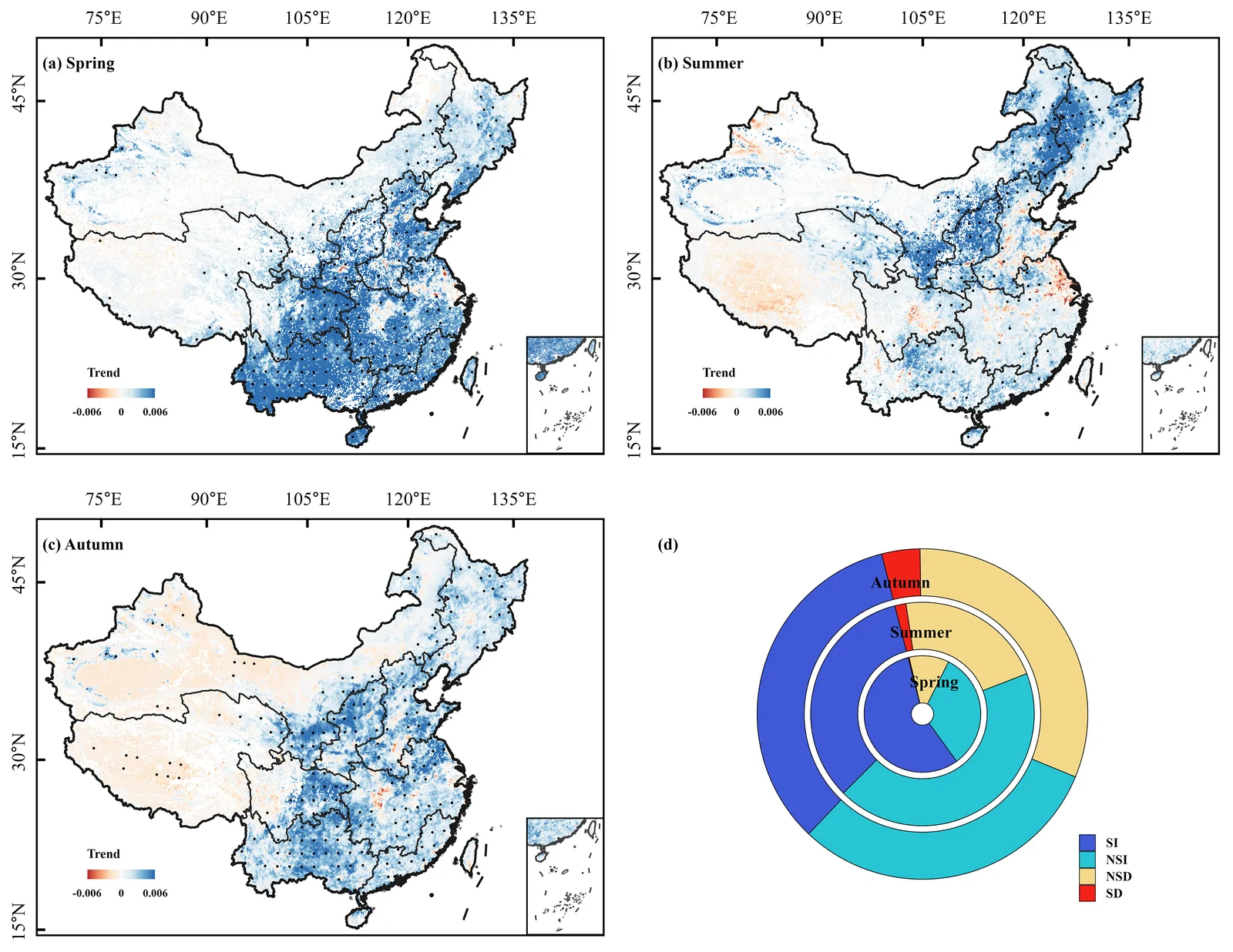
Spatial trends and area proportions of seasonal RSEI trend types in China from 2001 to 2022. Spatial trends of RSEI in **(a)** spring, **(b)** summer and **(c)** autumn. Black dots indicate significant trends (Mann-Kendall test, p < 0.05). **(d)** Area proportions of SI, NSI, NSD and SD trend types for spring, summer and autumn.

We analyzed the temporal trends of seasonal RSEI grid-by-grid from 2001 to 2022 using Sen’s slope and MK tests (**Figure 3**). In spring, 36.67% of the study area exhibited a statistically significant increase in RSEI (p < 0.05) (**Figure 3d**). These significantly increasing areas were mainly distributed in the YGP, SC, MYP, eastern SBR, HHP, LP, NCP, and the eastern and southern parts of the NAR (**Figure 3a**). Relatively stronger upward trends were concentrated in the YGP, southern LP, SC, western MYP, eastern SBR, and central HHP (**Figure 3a**). In contrast, 7.43% of the study area showed a decreasing spring RSEI trend, while only 0.20% exhibited a significant decrease.

In summer, 59.68% of the study area showed an increasing RSEI trend, of which 26.02% was statistically significant (**Figure 3d**). The significantly increasing areas were mainly concentrated in northern LP, northern NCP, SC, central YGP, and parts of northern HHP, as well as the eastern and southern margins of NAR (**Figure 3b**). In contrast, approximately 17.93% of the study area exhibited a decreasing summer RSEI trend, while only 1.26% showed a significant decrease, mainly scattered across eastern MYP (**Figure 3b**). In autumn, 56.68% of the study area showed an increasing RSEI trend, of which 29.70% was statistically significant (**Figure 3d**). The significantly increasing areas were mainly distributed in the YGP, SC, eastern SBR, northern LP, MYP, and HHP (**Figure 3c**). In contrast, the decreasing trend expanded to 30.76% of the study area, with 3.28% showing a statistically significant decline. These significantly declining areas were mainly located in western QTP and northern NAR (**Figures 3c, d**).

**Figure 4:**
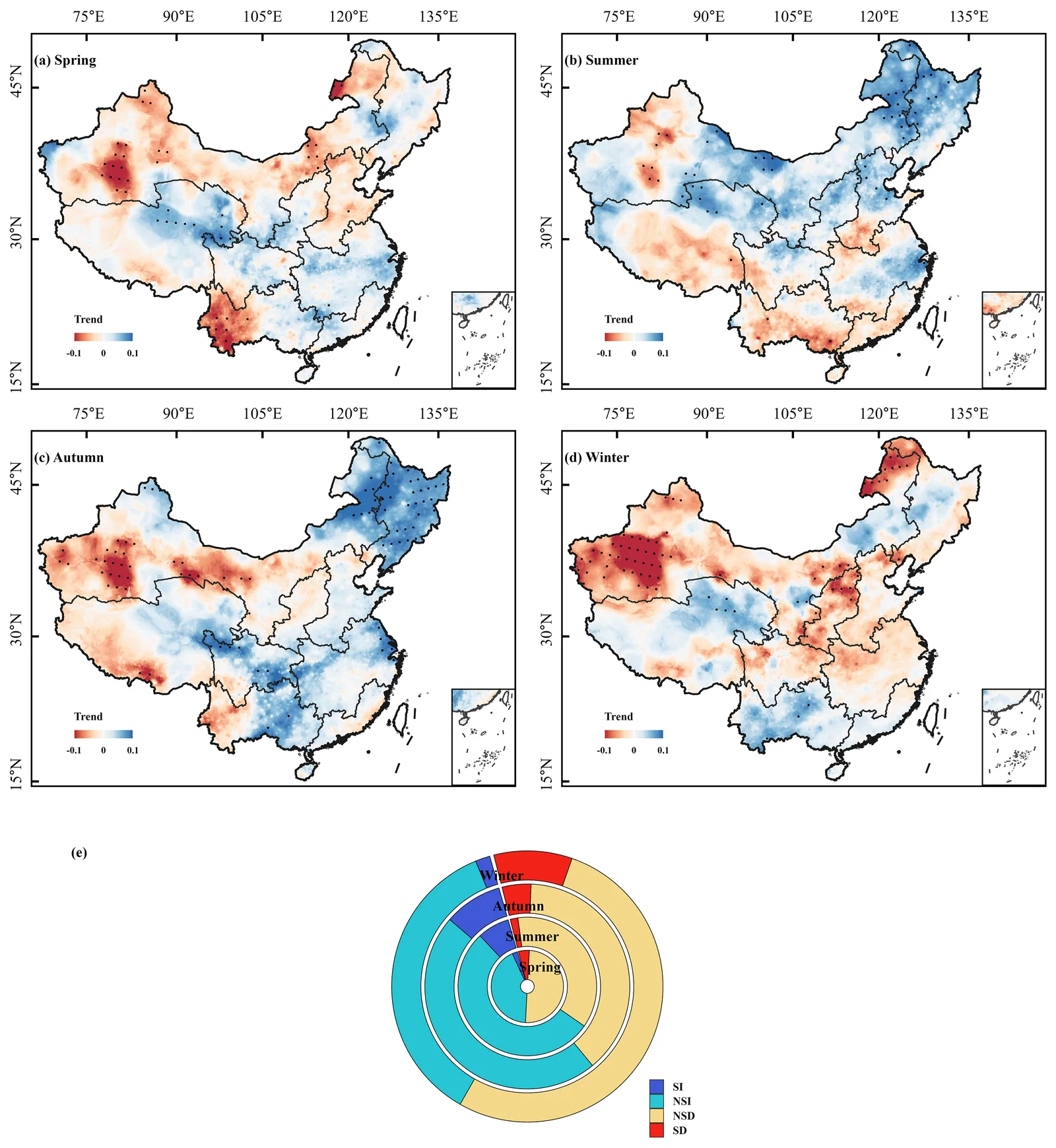
Spatial patterns and area proportions of seasonal SPEI trend types in China from 2001 to 2022. Spatial trends of SPEI in **(a)** spring, **(b)** summer, **(c)** autumn, and **(d)** winter. Black dots indicate significant trends based on the Mann-Kendall test (p < 0.05). **(e)** Area proportions of significant increase (SI), non-significant increase (NSI), non-significant decrease (NSD), and significant decrease (SD) trend types in spring, summer, autumn, and winter.

The seasonal trends of SPEI indicated that most regions exhibited increasing trends in summer and autumn (63.36% and 57.37%, respectively) (**Figure 4e**), which were mainly concentrated in the NCP, eastern NAR, and eastern MYP (**Figures 4b, c**). In contrast, regions with decreasing trends were mainly concentrated in the QTP and western NAR. In spring and winter, SPEI predominantly exhibited downward trends (56.46% and 64.09%) (**Figure 4e**), mainly in the NAR and the HHP, while upward trends were mainly observed in the eastern QTP and the central NCP (**Figures 4a, d**). In addition, the proportion of areas with a significant increase in SPEI was significantly higher in summer (10.32%) and autumn (8.42%) than in spring (2.36%) and winter (1.72%) (**Figure 4e**). In comparison, winter exhibited the highest proportion of significant decreases in SPEI (10.38%) (**Figure 4e**).

### 3.2 Concurrent and lagged effects of seasonal drought on ecological quality under climate change and human activities

**Figure 5:**
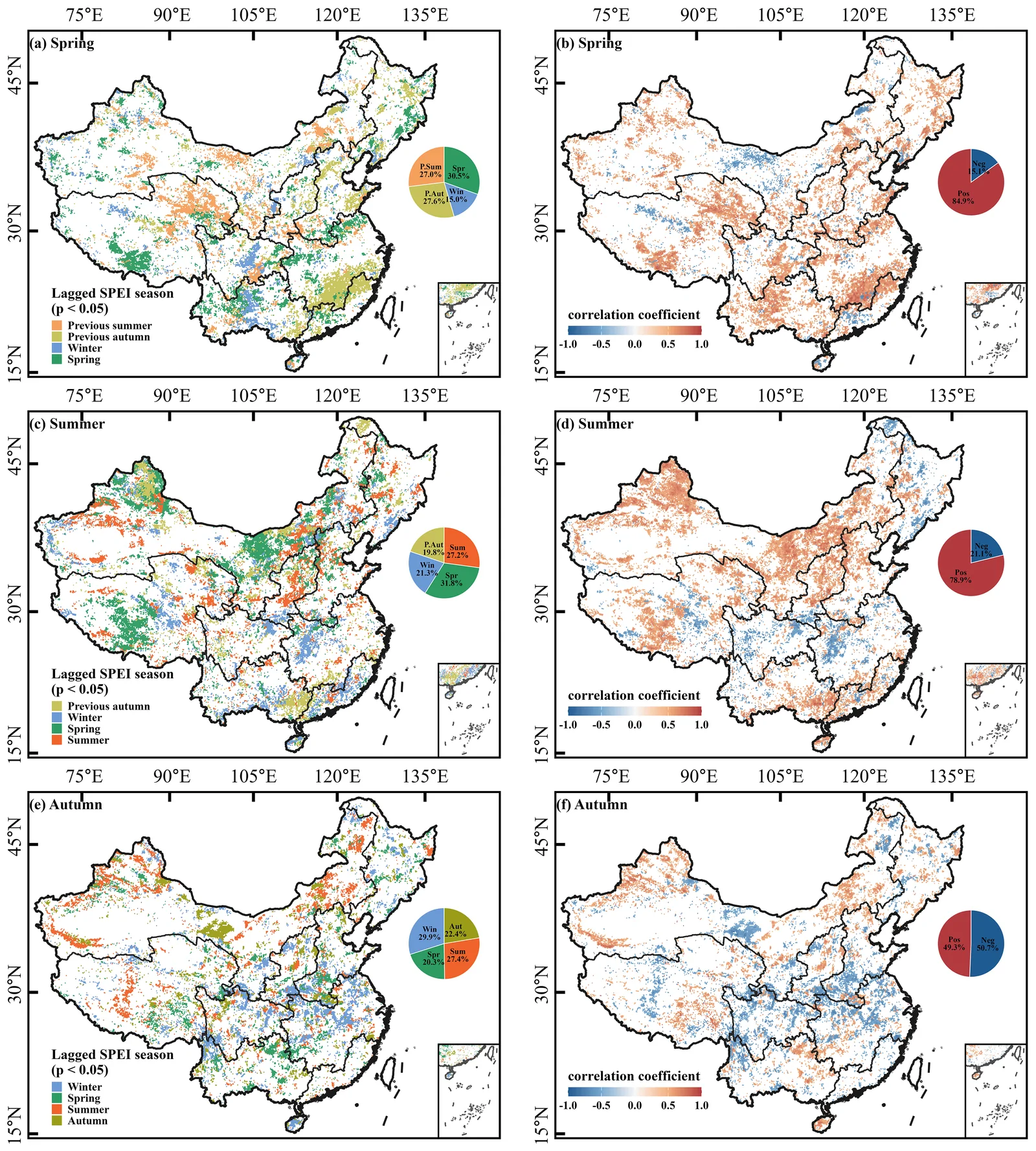
Spatial patterns of the dominant SPEI response season and corresponding partial correlation with seasonal RSEI in China. Spatial patterns of the dominant SPEI response season for **(a)** spring RSEI, **(c)** summer RSEI and **(e)** autumn RSEI. Spatial patterns of the corresponding maximum partial correlation coefficient between RSEI and SPEI for **(b)** spring, **(d)** summer and **(f)** autumn. Only pixels with significant partial correlations are shown (p < 0.05). Pie charts in **(a, c, e)** show the area proportions of different dominant SPEI response seasons, whereas pie charts in **(b, d, f)** show the area proportions of positive and negative partial correlations.

To assess the concurrent and lagged effects of seasonal drought on ecological quality, we calculated the pixel-wise partial correlations between seasonal RSEI and SPEI in different periods after controlling for air temperature, precipitation, solar radiation, soil moisture, and human activities. We then identified the drought season to which ecological quality showed the strongest response for each pixel. Collinearity diagnostics showed that the retained explanatory variables had acceptable levels of multicollinearity (**Supplementary Table 4**), supporting their joint use in the partial correlation models. Higher SPEI values indicate wetter moisture conditions, whereas lower SPEI values indicate more severe drought. A positive correlation between RSEI and SPEI indicates that wetter conditions favor improved ecological quality, whereas intensified drought leads to a decline in ecological quality. Overall, the area where seasonal RSEI showed a significant response to SPEI was largest in summer, accounting for 30.15% of the total study area, followed by spring (26.78%) and autumn (23.53%) (**Figures 5a, c, e**).

Among the pixels where spring RSEI showed a significant response to SPEI, the largest area corresponded to pixels with the highest absolute partial correlation coefficient with concurrent spring SPEI, accounting for 8.16% of the total study area and 30.48% of the spring RSEI significant response pixels (**Figure 5a**). The areas of pixels with the strongest partial correlations with SPEI in the previous autumn and previous summer were close to that corresponding to concurrent spring SPEI, accounting for 7.39% and 7.22% of the total study area, and 27.59% and 26.97% of the spring RSEI significant response pixels, respectively. By contrast, the area of pixels with the strongest partial correlation with SPEI in the preceding winter was relatively small, accounting for 4.01% of the total study area and 14.96% of the spring RSEI significant response pixels. Spatially, areas with the strongest partial correlation with SPEI in the previous summer were mainly distributed in parts of NAR and eastern QTP. Areas with the strongest partial correlation with SPEI in the previous autumn were mainly concentrated in southern MYP. Areas with the strongest partial correlation with SPEI in the preceding winter were mainly distributed in YGP and central SBR. Areas with the strongest partial correlation with concurrent spring SPEI were mainly distributed in eastern and southern NCP, southern QTP, and YGP (**Figure 5a**). The significant partial correlations between spring RSEI and SPEI were generally dominated by positive correlations, while negative correlation areas were only scattered in central-eastern NAR, western QTP, and parts of SC (**Figure 5b**).

Summer RSEI showed an evident seasonal lagged response to antecedent moisture conditions, with the response area corresponding to spring SPEI being the most extensive (**Figure 5c**). Specifically, among the pixels where summer RSEI showed a significant response, pixels with the highest absolute partial correlation coefficient with spring SPEI occupied the largest area, accounting for 9.58% of the total study area and 31.78% of the summer RSEI significant response pixels. Pixels with the strongest partial correlation with concurrent summer SPEI ranked second, accounting for 8.19% of the total study area and 27.16% of the summer RSEI significant response pixels. Pixels with the highest absolute partial correlation coefficients with SPEI in the preceding winter and previous autumn accounted for 6.41% and 5.97% of the total study area, and 21.25% and 19.81% of the summer RSEI significant response pixels, respectively. Spatially, areas with the strongest partial correlation with spring SPEI were mainly distributed in central and western NAR and western QTP. Areas with the strongest partial correlation with concurrent summer SPEI were scattered across NAR, parts of NCP, eastern QTP, and central LP (**Figure 5c**). The spatial distribution of the corresponding partial correlation coefficients showed that the significant partial correlations between summer RSEI and SPEI were generally dominated by positive correlations, whereas negative correlation areas were mainly distributed in central NCP, central QTP, SBR, and MYP (**Figure 5d**).

The response characteristics of autumn RSEI to SPEI differed markedly from those in spring and summer, with autumn ecological quality showing a more pronounced response to antecedent drought. Among the pixels where autumn RSEI showed a significant response, pixels with the highest absolute partial correlation coefficient with SPEI in the preceding winter occupied the largest area, accounting for 7.04% of the total study area and 29.92% of the autumn RSEI significant response pixels. Pixels with the highest absolute partial correlation coefficient with summer SPEI accounted for 6.44% of the total study area and 27.39% of the autumn RSEI significant response pixels. Pixels with the highest absolute partial correlation coefficients with concurrent autumn SPEI and spring SPEI accounted for 5.27% and 4.78% of the total study area, and 22.38% and 20.31% of the autumn RSEI significant response pixels, respectively (**Figure 5e**). Spatially, areas with the strongest partial correlation with SPEI in the preceding winter were mainly distributed in SBR, LP, MYP, and parts of YGP. Areas with the strongest partial correlation with summer SPEI were mainly distributed in parts of NAR and central-western QTP (**Figure 5e**). The spatial distribution of the corresponding partial correlation coefficients showed a clear interlaced pattern of positive and negative significant partial correlations between autumn RSEI and SPEI, with stronger spatial heterogeneity than in spring and summer (**Figure 5f**). Positive partial correlation pixels were distributed in central and western NAR, northern and central LP, eastern QTP, northern HHP, and local areas of SC. Negative partial correlation pixels were mainly found in central-western NAR, MYP, SBR, local areas of YGP, western QTP, and western HHP (**Figure 5f**).

**Figure 6:**
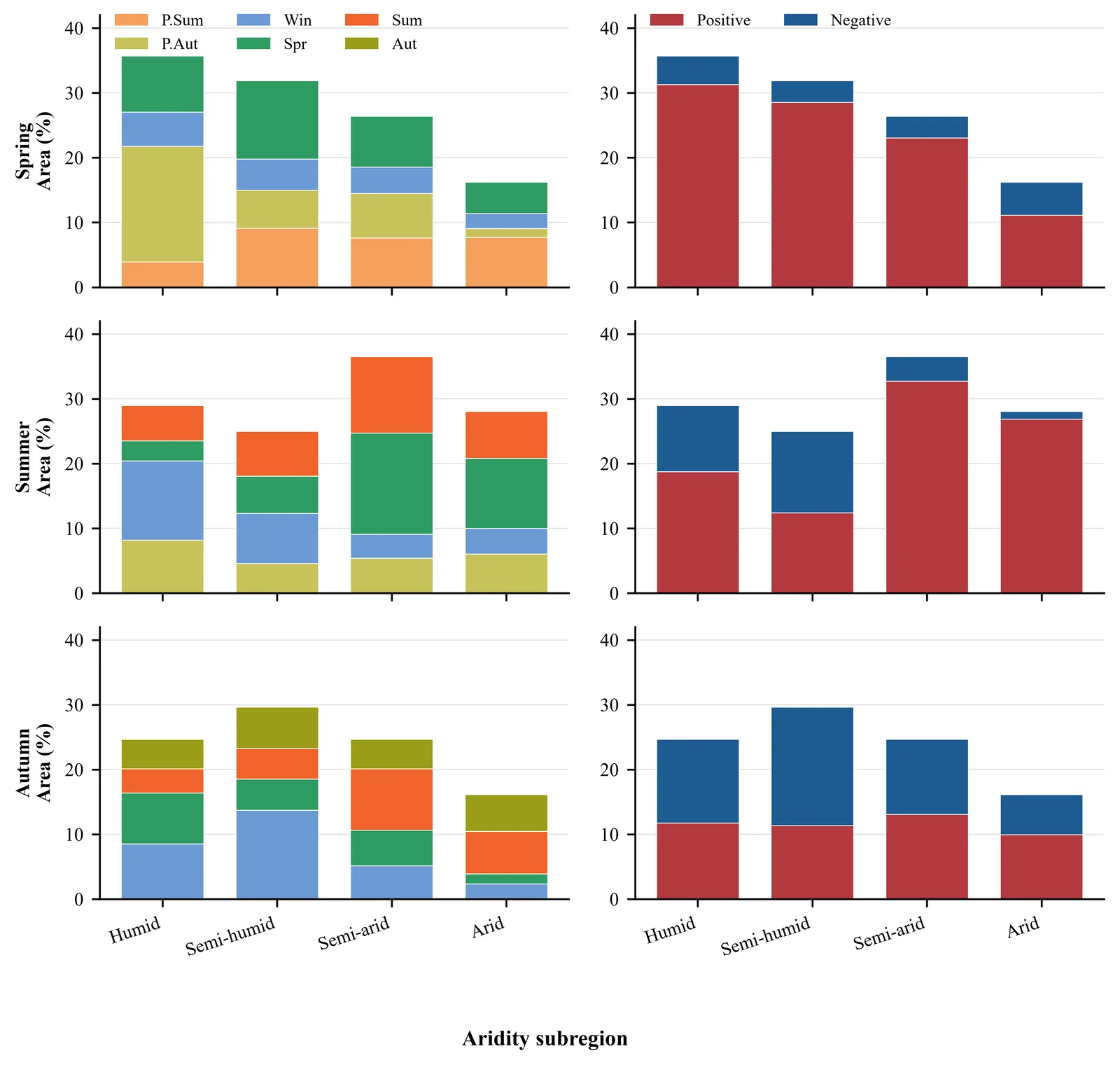
Area proportions of dominant SPEI response seasons and partial-correlation directions across aridity subregions. The left column shows the area proportions of the dominant SPEI response seasons for spring, summer and autumn RSEI within each aridity subregion. The right column shows the corresponding area proportions of positive and negative signed maximum partial correlations between RSEI and SPEI. P.Sum, previous summer; P.Aut, previous autumn; Win, winter; Spr, spring; Sum, summer; Aut, autumn. The area is filled in white due to the lack of data from SPEI in Taiwan Province.

In spring, the proportion of areas where RSEI showed a significant response to SPEI generally decreased with increasing regional aridity (**Figure 6**). Significant response pixels accounted for 35.70% of the humid zone, 31.89% of the semi-humid zone, and 26.41% of the semi-arid zone, but decreased to 16.25% in the arid zone. In terms of the SPEI season showing the strongest partial correlation with spring RSEI within each aridity zone, the humid zone had the largest pixel area corresponding to SPEI in the previous autumn, accounting for 17.85% of the zonal area. In both the semi-humid and semi-arid zones, concurrent spring SPEI corresponded to the largest pixel area, accounting for 12.08% and 7.85% of the respective zonal areas. In the arid zone, SPEI in the previous summer corresponded to the largest pixel area, accounting for 7.68% of the zonal area. The significant partial correlations in all aridity zones in spring were dominated by positive correlations. Among the significant response pixels in the humid, semi-humid, and semi-arid zones, the proportion of positive correlations exceeded 87%. The positive correlation proportion in the arid zone was relatively lower, at 68.58%.

In summer, the proportion of areas where RSEI showed a significant response to SPEI was highest in the semi-arid zone, with significant response pixels accounting for 36.51% of the zonal area, followed by the humid, arid, and semi-humid zones, accounting for 28.99%, 28.07%, and 24.96%, respectively (**Figure 6**). In terms of the SPEI season showing the strongest partial correlation with summer RSEI within each aridity zone, both the humid and semi-humid zones had the largest pixel areas corresponding to SPEI in the preceding winter, accounting for 12.24% and 7.71% of the respective zonal areas. In the semi-arid and arid zones, spring SPEI corresponded to the largest pixel areas, accounting for 15.64% and 10.82% of the respective zonal areas. Among the significant response pixels, the semi-arid and arid zones were both dominated by positive correlations, with positive correlation proportions reaching 89.73% and 95.76%, respectively. In the semi-humid zone, the proportions of positive and negative correlations were relatively similar, accounting for 49.64% and 50.36%, respectively.

In autumn, the proportion of areas where RSEI showed a significant response to SPEI was highest in the semi-humid zone, accounting for 29.63% of the zonal area (**Figure 6**). The significant response proportions in the humid and semi-arid zones were similar, accounting for 24.67% and 24.68%, respectively, while the arid zone showed the lowest proportion, at 16.15%. In terms of the SPEI season showing the strongest partial correlation with autumn RSEI within each aridity zone, both the humid and semi-humid zones had the largest pixel areas corresponding to SPEI in the preceding winter, accounting for 8.53% and 13.75% of the respective zonal areas. In the semi-arid and arid zones, summer SPEI corresponded to the largest pixel areas, accounting for 9.45% and 6.54% of the respective zonal areas. Compared with spring and summer, the direction of partial correlations in autumn showed more pronounced differences among aridity zones. Among the significant response pixels, the humid and semi-humid zones were dominated by negative correlations, with negative correlation proportions of 52.27% and 61.68%, respectively. In contrast, the semi-arid and arid zones were dominated by positive correlations, with positive correlation proportions of 53.00% and 61.75%, respectively.

### 3.3 Identification of dominant factors of seasonal RSEI considering lagged drought effects

**Figure 7:**
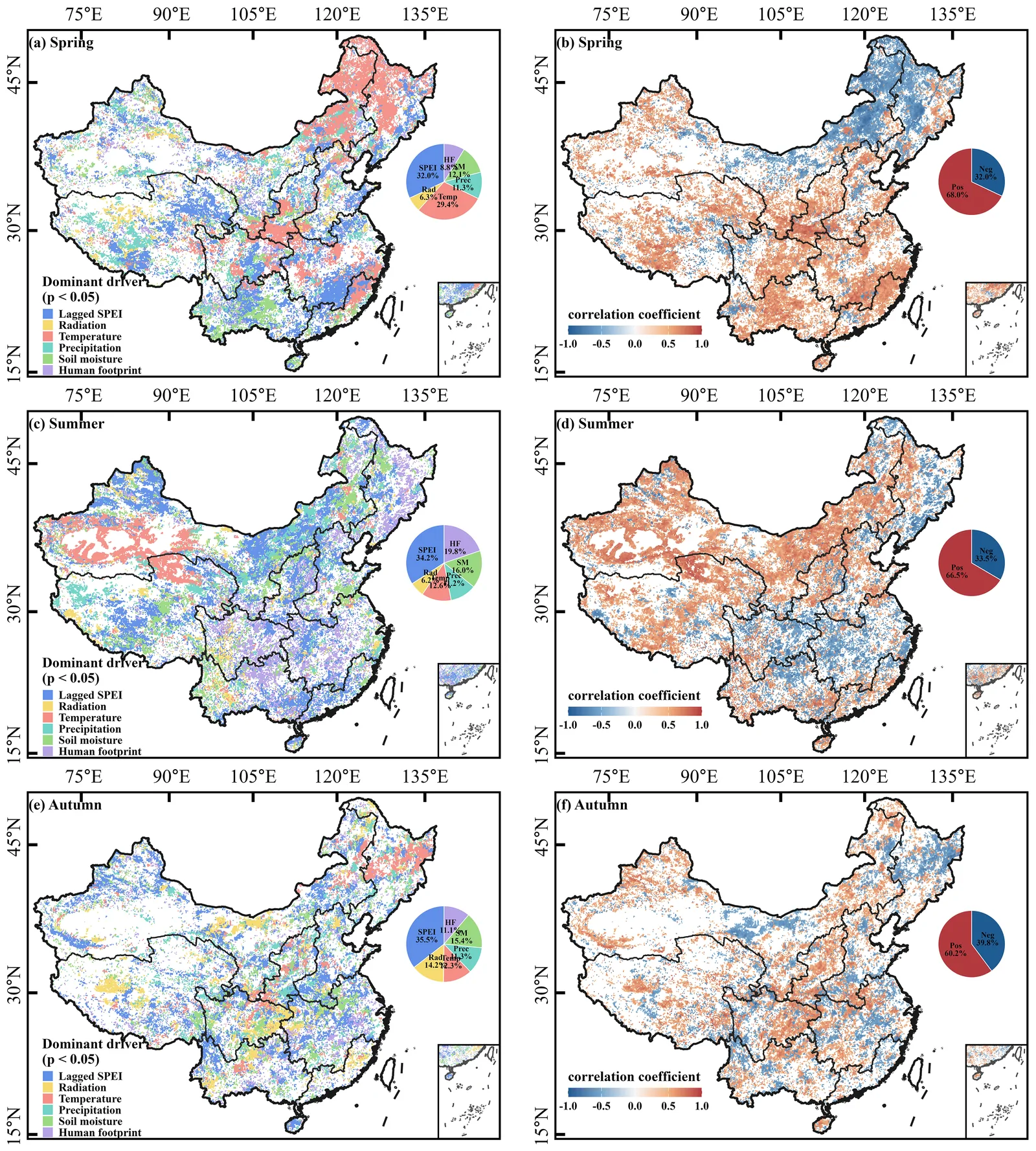
Spatial patterns of dominant factors and corresponding partial correlations for seasonal RSEI after accounting for SPEI response seasons. Spatial patterns of the dominant factors associated with **(a)** spring RSEI, **(c)** summer RSEI and **(e)** autumn RSEI after incorporating the dominant SPEI response season. Spatial patterns of the corresponding signed partial correlation coefficients for **(b)** spring, **(d)** summer and **(f)** autumn. Only pixels with significant partial correlations are shown (p < 0.05). Pie charts in **(a, c, e)** show the area proportions of dominant factors, whereas pie charts in **(b, d, f)** show the area proportions of positive and negative partial correlations. White color is due to the lack of data from SPEI in Taiwan Province.

After incorporating the concurrent and lagged effects of seasonal drought, we further compared the partial correlations between each environmental factor and seasonal RSEI. The factor with the largest absolute partial correlation coefficient and passing the significance test was identified as the dominant factor for each pixel (**Figure 7**).

In spring, SPEI was the dominant factor with the largest area, accounting for 32.0% of the significant dominant pixel area in spring; temperature was the second largest dominant factor, accounting for 29.4% (**Figure 7a**). Spatially, pixels for which SPEI was the dominant factor were relatively widely distributed, with clear occurrences in the eastern and southern QTP, YGP, central SBR, and the southern and northern parts of MYP. Pixels for which temperature was the dominant factor were mainly distributed in eastern NAR, northern NCP, and southern LP (**Figure 7a**). In summer, the dominant area of SPEI was the highest among the three seasons, accounting for 34.2% of the significant dominant pixel area in summer. In addition to SPEI, human footprint also showed a relatively large dominant area, accounting for 19.8% (**Figure 7c**). Spatially, pixels for which SPEI was the dominant factor were mainly distributed in central and western NAR, central QTP, LP, SC, and local areas of YGP. Pixels for which human footprint was the dominant factor mainly occurred in eastern NCP, MYP, SBR, northern HHP, central YGP, and local areas of SC (**Figure 7c**). In autumn, SPEI remained the dominant factor with the largest area, accounting for 35.5% of the significant dominant pixel area in autumn (**Figure 7e**). Spatially, pixels for which SPEI was the dominant factor were mainly distributed in eastern and western NAR, southern QTP, NCP, YGP, HHP, and local areas of SC (**Figure 7e**). Compared with spring and summer, the differences in dominant areas among other factors were relatively small in autumn. Soil moisture, radiation, temperature, precipitation, and human footprint accounted for 15.4%, 14.2%, 12.3%, 11.3%, and 11.1% of the significant dominant pixel area in autumn, respectively, and the significant dominant pixels were more dispersed among multiple factors (**Figure 7e**).

Overall, the significant dominant pixels in all three seasons were mainly characterized by positive correlations. Positive pixels accounted for 68.0%, 66.5%, and 60.2% of the significant dominant areas in spring, summer, and autumn, respectively, while negative pixels accounted for 32.0%, 33.5%, and 39.8%, respectively (**Figures 7b, d, f**). Among the three seasons, autumn showed a relatively higher proportion of negative pixels than spring and summer. After further distinguishing the direction of the dominant partial correlations, we calculated the proportions of positive and negative partial correlations for the two leading dominant factors in each season. In spring, pixels for which SPEI was the dominant factor were mainly characterized by positive partial correlations, with an internal positive proportion of 89.5%. By contrast, pixels for which temperature was the dominant factor were mainly characterized by negative partial correlations, with a negative proportion of 56.8%. In summer, pixels for which SPEI was the dominant factor were also mainly positively correlated, with a positive proportion of 82.1%; pixels for which human footprint was the dominant factor were mainly negatively correlated, with a negative proportion of 91.7%. In autumn, among pixels for which SPEI was the dominant factor, the proportions of positive and negative partial correlations were relatively similar, accounting for 53.3% and 46.7%, respectively. Pixels for which soil moisture was the dominant factor were mainly positively correlated, with a positive proportion of 88.4%.

**Figure 8:**
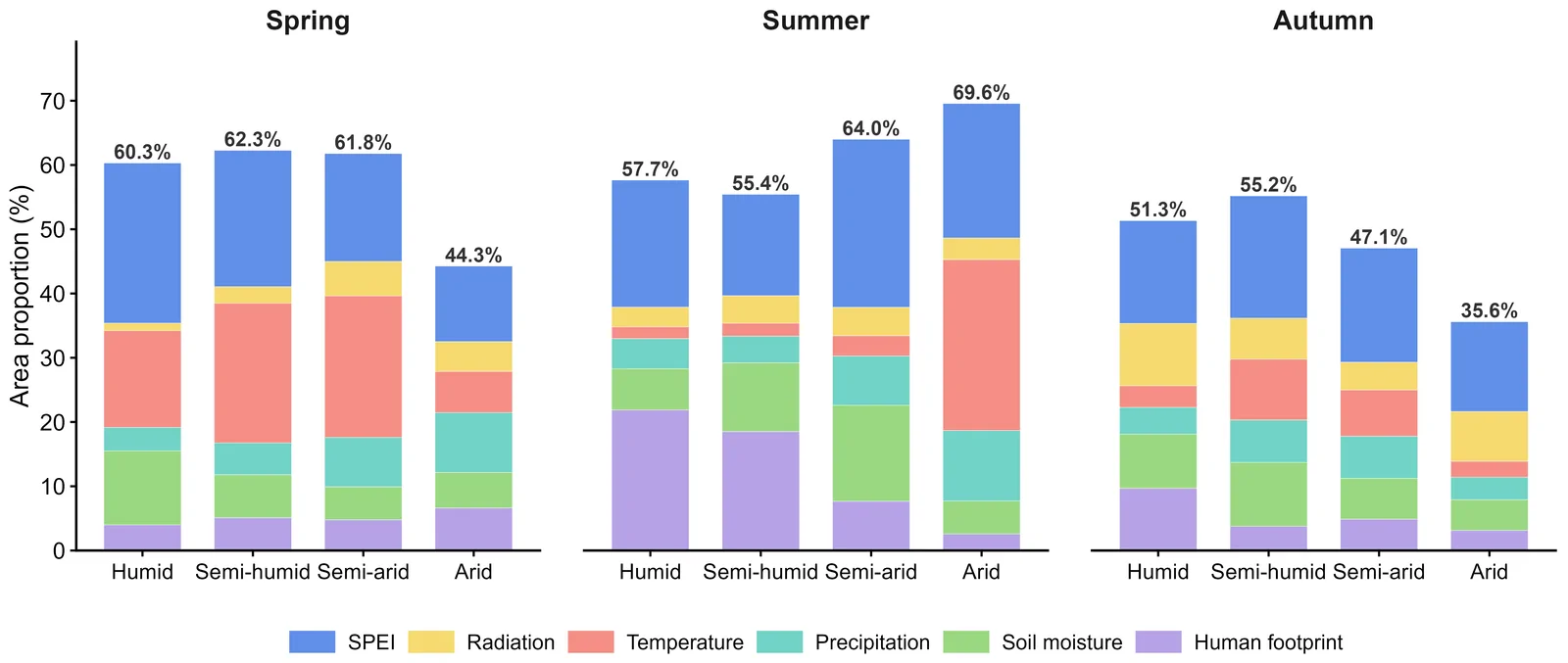
Area proportions of dominant factors associated with seasonal RSEI across aridity subregions. Area proportions of dominant factors associated with spring, summer and autumn RSEI across humid, semi-humid, semi-arid and arid subregions. Numbers above the bars indicate the total area proportion of grid cells with significant dominant-factor associations in each aridity subregion. SPEI, Standardized Precipitation Evapotranspiration Index.

To further examine whether the dominant-factor pattern of seasonal RSEI varied along the aridity gradient, we calculated the area proportion of each significant dominant factor within different aridity zones (**Figure 8**). The results showed clear differences in the dominant factors of RSEI among seasons and aridity zones. In spring, SPEI had the largest dominant area in the humid and arid zones, accounting for 24.9% and 11.8% of the corresponding zonal areas, respectively. In the semi-humid and semi-arid zones, temperature showed slightly larger dominant areas, accounting for 21.7% and 22.0%, respectively.

In summer, the composition of dominant factors differed more markedly among aridity zones. Human footprint had the largest dominant area in the humid and semi-humid zones, accounting for 21.9% and 18.5% of the corresponding zonal areas, respectively. In the semi-arid zone, SPEI was the primary dominant factor, with a dominant area proportion of 26.1%. In the arid zone, temperature was the dominant factor, accounting for 26.6% of the zonal area (**Figure 8**).

Compared with spring and summer, the dominant-factor pattern in autumn showed higher consistency across aridity zones. SPEI was the dominant factor with the largest spatial coverage in the humid, semi-humid, semi-arid, and arid zones, with dominant pixels accounting for 16.0%, 19.0%, 17.8%, and 14.0% of the corresponding zonal areas, respectively. In addition to SPEI, solar radiation and human footprint had relatively large dominant areas in the humid zone; soil moisture and temperature were more prominent in the semi-humid zone; and solar radiation was the secondary dominant factor in the arid zone (**Figure 8**).

## 4 Discussion

### 4.1 Spatiotemporal variation and characteristics of seasonal RSEI

Since 1999, China has implemented a series of ecological restoration projects aimed at restoring degraded ecosystems, including large-scale afforestation, the Grain for Green Program, and shelterbelt construction. These projects have provided an important background for the improvement of ecological environmental quality in China (Li et al., 2022; Deng et al., 2025). This study found that ecological quality in China generally improved in spring, summer, and autumn from 2001 to 2022. Similar patterns of ecological quality improvement have also been reported in previous national- and regional-scale studies (Zhang et al., 2023; Xin et al., 2024; Zhen et al., 2025). However, this improvement did not occur synchronously across different stages or regions, but showed clear seasonal differences and spatial heterogeneity.

Regionally, the improvement of ecological quality in LP and NCP may be related to the Grain for Green Program, the Three-North Shelterbelt Program, and long-term soil and water conservation measures (Yin et al., 2025). These measures help increase vegetation coverage, reduce bare land exposure, and improve land-surface ecological conditions (Li et al., 2022). The increase in RSEI in eastern and southern NAR and eastern HHP may be associated with the continuous implementation of national ecological restoration projects, cultivated land protection policies, and agricultural modernization measures (Li et al., 2023b). In addition, the Western Development Strategy and the regional coordinated development strategy may have improved ecological protection and environmental governance capacity in western China and surrounding regions, such as QTP and western NAR, to some extent (Yan et al., 2024). However, some areas within the study region still showed a decline in RSEI in summer and autumn, indicating that improvements resulting from ecological restoration and land management cannot fully offset the effects of regional climate variability and water limitation.

### 4.2 Different effects of seasonal drought on RSEI

In China, the impacts of seasonal drought on ecological quality show clear differences across climatic regions, seasons, and areas. The effects of drought on RSEI depend not only on drought conditions in the same season, but also on antecedent water availability. The response of spring RSEI to drought is closely related to both current-season water supply and antecedent moisture conditions. Antecedent climatic conditions can affect subsequent vegetation recovery and productivity through physiological legacy effects, root development, carbon storage, and phenological processes (Ding et al., 2020; Tang et al., 2024c). Spring is the stage of vegetation green-up and early canopy development, during which sufficient water supply can promote vegetation recovery and improve land-surface moisture conditions (Lian et al., 2024). Meanwhile, moisture conditions in the previous summer and autumn may continue to affect spring ecological quality through soil moisture memory, pre-winter vegetation status, and post-winter recovery processes (Li et al., 2023c). In water-limited and ecologically fragile regions such as NAR and QTP, antecedent drought may weaken the ecological foundation for spring recovery. By contrast, in parts of MYP, autumn and winter moisture conditions may play a more important role in regulating RSEI in the following spring, because these moisture conditions affect soil moisture recharge and vegetation recovery before the start of the next growing season (Du et al., 2025; Yang et al., 2026).

The relationship between summer drought and ecological quality was more significant than that in spring and autumn. This is mainly because summer corresponds to the peak vegetation growth season, when photosynthesis, transpiration, and canopy development are highly dependent on water supply (Schuldt et al., 2020; Tang et al., 2024a). Under high temperature and strong evaporative demand, drought can reduce transpiration, limit photosynthesis, decrease vegetation greenness, and intensify land-surface thermal and dryness conditions, all of which are directly reflected in the greenness, wetness, heat, and dryness components of RSEI (Zhang et al., 2016; Sun et al., 2023). The positive correlations between SPEI and RSEI in arid and semi-arid regions further indicate that, in water-limited areas, drought alleviation usually corresponds to improved ecological quality. This pattern was particularly evident in NAR, QTP, and LP, where sparse vegetation, grassland-dominated landscapes, or fragile alpine ecosystems make ecological quality more sensitive to seasonal water deficits (Jiang et al., 2023). Previous studies have also shown that vegetation greenness in arid regions is strongly regulated by water availability, and that increasing water limitation can enhance ecosystem sensitivity to drought (Zhang et al., 2016; Tang et al., 2024a; Lu et al., 2026).

The response of autumn ecological quality to seasonal drought was more complex. In water-limited regions, such as parts of NAR and QTP, higher SPEI generally indicates alleviated drought stress, which may help maintain vegetation greenness and land-surface moisture conditions in the late growing season. However, in humid and semi-humid regions, higher SPEI does not necessarily lead to higher RSEI. In regions such as MYP, SBR, and YGP, excessive moisture may be accompanied by increased cloud cover, reduced solar radiation, soil waterlogging, and changes in leaf development or autumn phenology, thereby weakening the positive effect of water supply on vegetation growth and ecological quality (Baldocchi et al., 2000; Chen et al., 2022; Li et al., 2024b). This may help explain why autumn RSEI showed positive SPEI associations in arid regions but weaker or negative associations in humid and semi-humid regions.

### 4.3 Driving factors of seasonal RSEI under drought and multi-factor influences

After incorporating the lagged effects of seasonal drought into the multifactor partial correlation analysis, SPEI was the dominant factor with the widest spatial distribution for spring, summer, and autumn RSEI. This result indicates that interannual changes in seasonal ecological quality cannot be explained only by concurrent precipitation, temperature, or soil moisture, and that the drought background jointly shaped by water supply and atmospheric evaporative demand is of great importance (Bastos et al., 2020; Chen et al., 2025).

Spring RSEI was mainly jointly constrained by seasonal drought and temperature. Spring represents the transition stage from vegetation dormancy to growth recovery, during which ecological quality changes depend not only on antecedent moisture conditions but also on thermal conditions (Hao et al., 2024; Huang and Zhai, 2024). Vegetation responses to moisture conditions are jointly regulated by temperature, solar radiation, soil moisture, and phenological processes, and spring phenology can also affect the rate of ecosystem recovery from drought (Wei et al., 2023; Ge et al., 2024). In low-temperature or energy-limited regions, warming may promote vegetation green-up and early growth. However, in relatively water-limited or agro-pastoral transition regions such as NAR, LP, and northern NCP, warming may also intensify water stress by increasing evaporative demand (Wang et al., 2025).

The response of summer RSEI to seasonal drought was more pronounced, which is related to the fact that summer corresponds to the peak vegetation growth season, when both evaporative demand and water consumption are high (Wu et al., 2022; Huang and Zhai, 2024). Meanwhile, human footprint was the secondary factor affecting summer ecological quality and dominated parts of NCP, MYP, SBR, YGP, and SC. These regions generally have higher cropland use intensity, urban construction intensity, population density, and economic development levels than NAR and QTP. Cropping systems and irrigation management can affect vegetation growth status and land-surface moisture conditions (Xin et al., 2024), expansion of construction land and bare land exposure can increase the dryness and heat components, and land cover change directly alters the greenness component (Qin et al., 2024). Therefore, in these regions, human footprint had greater explanatory power for ecological quality than climatic factors.

The dominant factors of autumn RSEI were more complex than those in spring and summer. Although SPEI remained the primary factor, the importance of soil moisture and solar radiation became more evident, and the proportions of positive and negative partial correlations for SPEI were more similar. In semi-arid and arid regions, higher SPEI usually indicates weakened water stress, which is conducive to maintaining vegetation greenness and land-surface moisture (Zhang and Zhang, 2019). However, in humid and semi-humid regions, excessive moisture may be accompanied by higher soil moisture, insufficient radiation, or increased cloudy and rainy weather, thereby weakening vegetation photosynthetic conditions. Positive responses are more common in water-limited regions, whereas weaker or negative responses may occur in humid regions, which is related to water surplus, radiation limitation, and ecosystem resistance (Qiao et al., 2024).

### 4.4 Policy suggestions

The response of ecological quality to drought showed clear seasonal differences and lagged characteristics. Therefore, ecological conservation and drought risk management should not be formulated solely on the basis of annual-scale or concurrent drought conditions. Antecedent and current-season moisture conditions are important for spring ecological recovery. For eastern and southern QTP, YGP, central SBR, and parts of MYP, spring ecological quality assessment should incorporate moisture conditions in the previous autumn and winter to identify the risk of water deficits before the green-up period (Piao et al., 2019). For regions where temperature is the dominant influencing factor, such as eastern NAR, northern NCP, and southern LP, warming may simultaneously promote green-up and enhance evapotranspiration (Lu et al., 2026). Therefore, vegetation restoration should not simply pursue high coverage or high-density afforestation, but should select drought-tolerant vegetation according to water resource carrying capacity, control restoration density, and strengthen soil moisture conservation and soil and water conservation measures (Liu et al., 2023).

Summer policy priorities should distinguish between water-limited regions and regions with high human activity intensity. Water conditions are an important factor in summer ecological quality changes, while human activities are a secondary factor. In water-limited regions such as NAR, QTP, and LP, priority should be given to strengthening water security during the peak growing season, grassland grazing management, and early drought warning. In eastern NCP, MYP, SBR, northern HHP, central YGP, and local areas of SC, greater attention should be paid to the effects of cropland use intensity, urban expansion, construction land increase, and agricultural management practices on summer RSEI (Li et al., 2023a). For these regions dominated by human footprint, ecological management should not attribute ecological quality changes only to climatic drought, but should incorporate land-use intensity, irrigation and drainage systems, bare land exposure, and ecological space occupation into summer ecological quality regulation (Raimundo et al., 2018; Gao et al., 2020).

Autumn ecological management should avoid the assumption that all water-increasing strategies are beneficial to ecological quality. Autumn drought remained the dominant factor affecting the largest area in China, but its positive and negative effects coexisted and covered comparable areas. Compared with spring and summer, autumn ecological quality more strongly reflected the joint regulation of water and energy conditions. In arid and semi-arid regions, attention should be focused on the effects of summer and antecedent winter water deficits on vegetation maintenance in the late growing season (Chen et al., 2021; Liu et al., 2025). In particular, in regions such as NAR, QTP, and LP, soil moisture monitoring before autumn and stability assessment of ecological restoration should be strengthened. By contrast, in humid and semi-humid regions, such as MYP, SBR, SC, and YGP, attention should also be paid to the effects of excessive soil moisture, insufficient solar radiation, waterlogging, and autumn phenological changes on RSEI (Qiao et al., 2024). Therefore, autumn management in these regions should shift from drought resistance alone to the integrated consideration of drought and waterlogging, drainage regulation, and farmland moisture management.

### 4.5 Limitations and future work

Several limitations should be acknowledged. First, the Human Footprint dataset provides an annual indicator of anthropogenic pressure. Therefore, the same annual value was used for spring, summer, and autumn within each year, which may not fully capture seasonal variations in land management, irrigation, construction activity, and other human disturbances. Second, this study focused on SPEI-3 to represent seasonal drought. Drought processes operating at shorter or longer accumulation timescales, such as SPEI-1, SPEI-6, and SPEI-12, were not systematically compared. Future studies could examine the sensitivity of seasonal RSEI to multiple SPEI accumulation timescales. Third, the statistical framework was based on partial correlation analysis. Although this approach can identify conditional associations after controlling for selected climatic and anthropogenic variables, it cannot establish direct causal pathways. Future work could incorporate structural equation modelling, machine-learning attribution, or ecosystem process models to further test the pathways linking drought, hydrothermal conditions, human activities, and ecological quality. Finally, this study used fixed calendar seasons for national-scale comparison. However, phenological stages vary across aridity zones, vegetation types, and elevation gradients. Future studies could combine phenological observations or region-specific growing-season definitions to improve the ecological interpretation of seasonal RSEI responses.

## 5 Conclusions

This study reconstructed spring, summer, and autumn RSEI datasets for China from 2001 to 2022 and combined them with seasonal SPEI, climatic variables, and human activity data to investigate the seasonal effects of drought on ecological quality and their differences among dry and wet regions. The results showed that ecological quality in China generally improved in spring, summer, and autumn during the study period, but the improvement differed among seasons and regions. Spring showed the fastest increase and the largest area of significant improvement, whereas autumn had a wider area of decline. Therefore, ecological quality improvement in China was not spatially or seasonally uniform. Seasonal drought had different effects on RSEI in spring, summer, and autumn. Summer RSEI showed the largest significant response area to SPEI and was mainly affected by spring drought, while spring RSEI was mainly affected by concurrent spring drought. Autumn RSEI was more strongly affected by winter and summer droughts, and ecological quality in the late growing season was jointly influenced by earlier water conditions and growing season drought stress. The drought effects also varied among dry and wet regions. In spring, the significant response area generally decreased with increasing dryness, whereas in summer it was highest in semi-arid regions. In autumn, drought effects were mainly positive in semi-arid and arid regions but negative in humid and semi-humid regions. SPEI was the dominant factor with the widest significant area in spring, summer, and autumn RSEI. Other factors also showed clear seasonal differences: temperature was more important in spring, human footprint was more prominent in summer in areas with stronger agricultural and urban development, and soil moisture, solar radiation, temperature, precipitation, and human footprint showed more dispersed effects in autumn. Seasonal ecological quality monitoring and drought risk management should consider the season of drought occurrence, lagged drought effects, and differences among dry and wet regions, rather than relying only on annual drought conditions or concurrent drought effects.

## Data Availability

The original contributions presented in the study are included in the article/**Supplementary Material**. Further inquiries can be directed to the corresponding author.
