# Supplementary material for: Seasonal ecological quality in China: concurrent and lagged drought responses and dominant factors
Source: Front Plant Sci. 2026 Aug 24;17:1850459. doi: 10.3389/fpls.2026.1850459 (PMC13547510; doi:10.3389/fpls.2026.1850459)
Supplement: Supplementary file 1 [file Table1.docx]

Supplementary Material

This supplementary material provides detailed information on the MODIS preprocessing, water masking, seasonal compositing, component-indicator calculation, normalization, and PCA-based integration procedures used to construct the seasonal Remote Sensing Ecological Index (RSEI). It further documents the PCA implementation and annual diagnostics, including PC1 contribution rates, loading directions, ecological orientation correction, and annual comparability checks. Detailed procedures for the pixel-wise Mann–Kendall (MK) trend test, Sen’s slope estimation, and collinearity diagnostics for the partial-correlation models are also provided.

# 1 Remote Sensing Data Preprocessing for RSEI Construction

Before index construction, quality control was performed separately for different MODIS products. For MOD09A1, the StateQA band was used to remove pixels affected by clouds, cloud shadows, and cirrus clouds, and only pixels with uncontaminated cloud state, cloud shadow state, and cirrus state were retained. For MOD13A1, the SummaryQA band was used for quality screening, and pixels with SummaryQA values less than or equal to 1 were retained to remove the effects of clouds, snow, and ice. For MOD11A2, the basic quality control information from the QC_Day band was used, and daytime land surface temperature pixels with good or acceptable quality were retained. To maintain temporal consistency with seasonal SPEI-3, spring, summer, and autumn RSEI were constructed from MODIS images in March-May, June-August, and September-November of each year, respectively. For each season-year combination, the quality-controlled MOD13A1, MOD11A2, and MOD09A1 images were composited using the median method to obtain NDVI, LST, and surface reflectance images for the corresponding season.

# 2 Calculation of Component Indicators

The RSEI is composed of four indicators representing greenness, humidity, heat, and dryness, respectively (Xu, 2013). All indicators were derived from MODIS products (Table 1 in the main text).

(1) Greenness (NDVI)

The normalized difference vegetation index (NDVI) can effectively reflect vegetation growth and vegetation cover. The formula is as follows:

$NDVI=\frac{\rho_{NIR}-\rho_{Red}}{\rho_{NIR}+\rho_{Red}}$ (1)

Where $\rho_{NIR}$ and $\rho_{Red}$ are the reflectance of the infrared band and near-infrared band, respectively.

(2) Heat (LST)

LST was derived from the MOD11A2 LST_Day_1km layer using the scale factor of 0.02 and converted from Kelvin to Celsius:

$LST=0.02\times DN_{S}-273.15$ (2)

where $DN_{S}$ is the gray value of the land surface temperature image.

(3) Humidity (WET)

WET is derived from tasseled cap transformation and can accurately reflect soil and vegetation moisture information. The formula is as follows:

$\begin{matrix} WET=0.1147\times RED+0.2489\times NIR1+0.2408\times BLUE+0.3132\times GREEN-0.3122\times NIR2 \\ -0.6416\times SWIR1-0.5087\times SWIR2 \end{matrix}$ (3)

where *RED*, *NIR1*, *BLUE*, *GREEN*, *NIR2*, *SWIR1* and *SWIR2* are the red, near infrared 1, blue, green, near infrared 2, short wave infrared 1 and shortwave infrared 2 band reflectance of MOD09A1, respectively.

(4) Dryness (NDBSI)

Dryness, expressed by the normalized difference built-up and soil index (NDBSI), indicates the amounts of built-up areas. The formula is as follows:

$NDBSI=\frac{SI+BI}{2}$ (4)

$SI=\frac{(SWIR1+RED)-(NIR+BLUE)}{(SWIR1+RED)+(NIR+BLUE)}$ (5)

$IBI=\frac{2\times SWIR1/(SWIR1+RED)-(NIR1/(NIR1+RED)+GREEN/(GREEN+SWIR1))}{2\times SWIR1/(SWIR1+RED)+(NIR1/(NIR1+RED)+GREEN/(GREEN+SWIR1))}$ (6)

where *SI* is the soil index, and *IBI* is the urban building index. *RED*, *BLUE*, *GREEN*, *NIR1*, *NIR2*, and *SWIR1* are the reflectance of MOD09A1 in the red, blue, green, near-infrared 1, near-infrared 2, and short-wavelength infrared 1 bands, respectively.

(5) Normalization

Due to the different dimensions of these four indices, they were normalized before principal component analysis (PCA). The formula is as follows:

$\text{N}_{\text{i}}=\frac{I_{i}-I_{\min}}{I_{\max}-I_{\min}}$ (7)

where *N_i_* represents the normalized value of a given indicator; *I_i_* represents the value of the indicator; *I_min_* and *I_max_* represent the minimum and maximum values of the indicator, respectively.

# 3 Water masking based on MNDWI

To reduce the interference of large water bodies with WET, LST, index normalization, and principal component analysis, a water body mask was constructed based on the MOD09A1 surface reflectance product. First, the Modified Normalized Difference Water Index (MNDWI) was calculated for each MOD09A1 image that passed StateQA quality control (Xu, 2013):

$MNDWI=\frac{Green-SWIR1}{Green+SWIR1}$	(8)

where Green and SWIR1 correspond to the sur_refl_b04 and sur_refl_b06 bands in the MOD09A1 product, respectively. The MNDWI images calculated for all scenes within the corresponding season were then composited using the mean method to obtain the seasonal MNDWI image. Pixels with MNDWI values lower than 0.2 were defined as non-water pixels and retained, whereas pixels with MNDWI values greater than or equal to 0.2 were identified as water bodies and uniformly removed from the subsequent calculations of NDVI, LST, WET, and NDBSI.

# 4 PCA procedure for seasonal RSEI construction

Principal component analysis (PCA) was used to integrate the four normalized ecological indicators into a single RSEI component while reducing redundancy among the indicators. PCA was performed using an orthogonal linear transformation based on the covariance matrix of the four normalized indicators (Xu, 2013; Li et al., 2023). For each season-year combination, the normalized NDVI, WET, LST, and NDBSI layers were first stacked into a data matrix $X$, in which each row represented a valid non-water pixel and each column represented one ecological indicator.

The principal component score of pixel $i$on principal component $j$was calculated as:

$PC_{\left( i,j \right)}=\sum_{\left( k=1 \right)}^{4} X_{\left( i,k \right)}E_{\left( k,j \right)}$	(9)

where $PC_{i,j}$ is the $j$-th principal component score of pixel $i$; $X_{i,k}$ is the normalized value of the $k$-th ecological indicator for pixel $i$; and $E_{k,j}$ is the element of the eigenvector matrix corresponding to the $k$-th indicator and the $j$-th principal component.

The eigenvectors and eigenvalues were obtained from the covariance matrix of the normalized indicators:

$C=E\Lambda E^{T}$	(10)

where $C$ is the covariance matrix of the normalized ecological indicators, $E$ is the eigenvector matrix, $E^{T}$is the transpose of the eigenvector matrix, and $\Lambda$is the diagonal matrix of eigenvalues. The eigenvalue of each principal component represents the amount of variance explained by that component. The contribution rate of PC1 was calculated as:

$C_{PC1}=\frac{\lambda_{1}}{\sum_{i=1}^{4} \lambda_{i}}\times100\%$	(11)

where $C_{PC1}$is the contribution rate of the first principal component, $\lambda_{1}$is the eigenvalue of PC1, and $\lambda_{i}$is the eigenvalue of the $i$-th principal component.

PC1 was used to construct RSEI because it explained the largest proportion of the total variance among the four ecological indicators. Since the sign of PCA loadings is mathematically arbitrary, the ecological direction of PC1 was checked for each season-year combination. Higher ecological quality should correspond to higher NDVI and WET and lower LST and NDBSI. Therefore, PC1 was oriented so that NDVI and WET had positive loadings, whereas LST and NDBSI had negative loadings. If the loading direction was reversed, the PC1 scores were multiplied by -1 before RSEI normalization.

After direction correction, PC1 was normalized to the range of 0-1 to obtain the final RSEI:

$\mathrm{RSE}I_{i}=\frac{\mathrm{PC}1_{i}-PC1_{\min}}{\mathrm{PC}1_{\max}-PC1_{\min}}$	(12)

where $\mathrm{RSE}I_{i}$is the RSEI value of pixel $i$, $\mathrm{PC}1_{i}$is the direction-corrected PC1 score, and $\mathrm{PC}1_{\min}$and $\mathrm{PC}1_{\max}$are the minimum and maximum PC1 values among valid pixels for the corresponding season-year combination.

In this study, PCA was conducted independently for each season-year combination rather than applying a fixed PCA model to all years. Therefore, we evaluated the annual comparability of the seasonal RSEI by examining the PC1 contribution rate, and loading directions of NDVI, WET, LST, and NDBSI for each year and season. The PCA diagnostic results are reported in Tables S1-S3. The consistently positive NDVI and WET loadings and negative LST and NDBSI loadings indicate that PC1 maintained a stable ecological meaning across years and seasons, supporting its use for seasonal RSEI construction and subsequent spatiotemporal analysis.

# 5 Mann–Kendall Trend Test and Sen’s Slope Estimator

Mann–Kendall Trend Test and Sen's slope estimation method is expressed as follows (Mann, 1945; Sen, 1968; Kendall, 1975; Hirsch et al., 1982):

$Z=\left\{\begin{matrix} \frac{S-1}{\sqrt{\text{Var}(S)}}, & S>0 & \\ 0, & S=0 & \\ \frac{S+1}{\sqrt{\text{Var}(S)}}, & S<0 & \end{matrix}\right.$ (13)

$\text{Var}(S)=\frac{n(n-1)(2n+5)}{18}$ (14)

$S=\sum_{i=1}^{n-1} \sum_{j=i+1}^{n} \left\{\begin{matrix} 1, & \text{if }x_{j}-x_{i}>0 & \\ 0, & \text{if }x_{j}-x_{i}=0 & \\ -1, & \text{if }x_{j}-x_{i}<0 & \end{matrix}\right.$ (15)

$\beta=Median\left(\frac{X_{j}-X_{i}}{j-i}\right),n>j>i>0$ (16)

where $Z$ is the standardized Mann–Kendall (MK) test statistic, $\text{Var}\left( S \right)$ is the variance of $S$, $\beta$ denotes the trend degree of the sequence, *n* is the length of the time series. $Median$ represents the median values; *i* and *j* are the *i*th and *j*th positions of the time series, respectively; $X_{j}$ and $X_{i}$ are the pixel values of the *i*th and *j*th RSEI or SPEI, respectively.

**Table S1. Annual PCA diagnostics for spring RSEI construction from 2001 to 2022.**

| **Year** | **PC1 contribution (%)** | **NDVI loading** | **WET loading** | **LST loading** | **NDBSI loading** |
| --- | --- | --- | --- | --- | --- |
| 2001 | 65.31 | 0.81342 | 0.50417 | -0.28943 | -0.01992 |
| 2002 | 70.90 | 0.86987 | 0.45898 | -0.18064 | -0.00594 |
| 2003 | 65.52 | 0.87906 | 0.46069 | -0.12249 | -0.00335 |
| 2004 | 66.13 | 0.82203 | 0.46469 | -0.32913 | -0.00218 |
| 2005 | 63.06 | 0.84173 | 0.47197 | -0.25960 | -0.03656 |
| 2006 | 65.01 | 0.84765 | 0.50083 | -0.17501 | -0.00469 |
| 2007 | 68.21 | 0.83755 | 0.47417 | -0.27045 | -0.02295 |
| 2008 | 67.72 | 0.86186 | 0.47117 | -0.18758 | -0.00192 |
| 2009 | 70.69 | 0.85325 | 0.45315 | -0.25811 | -0.00011 |
| 2010 | 66.49 | 0.84522 | 0.45802 | -0.27208 | -0.04241 |
| 2011 | 66.52 | 0.85677 | 0.43619 | -0.27494 | -0.00922 |
| 2012 | 66.46 | 0.89456 | 0.39344 | -0.21190 | -0.00799 |
| 2013 | 66.71 | 0.88402 | 0.39230 | -0.25282 | -0.02630 |
| 2014 | 70.72 | 0.87363 | 0.43973 | -0.20831 | -0.00314 |
| 2015 | 69.18 | 0.87292 | 0.43438 | -0.22195 | -0.00779 |
| 2016 | 71.55 | 0.88776 | 0.40199 | -0.22407 | -0.00879 |
| 2017 | 69.40 | 0.88648 | 0.40221 | -0.22832 | -0.01586 |
| 2018 | 72.07 | 0.85668 | 0.42116 | -0.29763 | -0.01192 |
| 2019 | 68.68 | 0.88535 | 0.39086 | -0.25178 | -0.00168 |
| 2020 | 71.19 | 0.89716 | 0.38963 | -0.20689 | -0.02210 |
| 2021 | 70.89 | 0.91370 | 0.38318 | -0.13456 | -0.01443 |
| 2022 | 75.14 | 0.92341 | 0.38284 | -0.01716 | -0.02148 |

Note: PCA was performed independently for each spring season-year combination (March-May).

**Table S2. Annual PCA diagnostics for summer RSEI construction from 2001 to 2022.**

| **Year** | **PC1 contribution (%)** | **NDVI loading** | **WET loading** | **LST loading** | **NDBSI loading** |
| --- | --- | --- | --- | --- | --- |
| 2001 | 82.42 | 0.90581 | 0.30063 | -0.29609 | -0.03828 |
| 2002 | 82.25 | 0.91840 | 0.28917 | -0.27004 | -0.00003 |
| 2003 | 82.23 | 0.92849 | 0.28889 | -0.23320 | -0.00844 |
| 2004 | 84.66 | 0.92758 | 0.30600 | -0.21439 | -0.00007 |
| 2005 | 83.68 | 0.93506 | 0.23960 | -0.26090 | -0.01327 |
| 2006 | 83.31 | 0.91012 | 0.31064 | -0.27345 | -0.02021 |
| 2007 | 81.85 | 0.91328 | 0.29583 | -0.28001 | -0.00102 |
| 2008 | 82.01 | 0.92165 | 0.25879 | -0.28572 | -0.04413 |
| 2009 | 83.79 | 0.91102 | 0.29333 | -0.28978 | -0.00536 |
| 2010 | 84.81 | 0.93223 | 0.27447 | -0.23578 | -0.00390 |
| 2011 | 80.86 | 0.90624 | 0.29990 | -0.29246 | -0.05711 |
| 2012 | 83.75 | 0.93810 | 0.25521 | -0.23351 | -0.01740 |
| 2013 | 84.32 | 0.91370 | 0.28598 | -0.28841 | -0.01352 |
| 2014 | 85.16 | 0.92733 | 0.29452 | -0.23067 | -0.01059 |
| 2015 | 83.41 | 0.92024 | 0.28431 | -0.26892 | -0.00173 |
| 2016 | 82.78 | 0.92115 | 0.27346 | -0.27665 | -0.01284 |
| 2017 | 80.81 | 0.93136 | 0.25581 | -0.25870 | -0.01436 |
| 2018 | 83.52 | 0.93349 | 0.26500 | -0.24148 | -0.00778 |
| 2019 | 82.61 | 0.91878 | 0.30323 | -0.25236 | -0.01454 |
| 2020 | 83.47 | 0.91795 | 0.25919 | -0.30031 | -0.00121 |
| 2021 | 83.55 | 0.91649 | 0.31040 | -0.25238 | -0.00208 |
| 2022 | 84.26 | 0.94512 | 0.31006 | -0.10278 | -0.00676 |

Note: PCA was performed independently for each summer season-year combination (June-August).

**Table S3. Annual PCA diagnostics for autumn RSEI construction from 2001 to 2022.**

| **Year** | **PC1 contribution (%)** | **NDVI loading** | **WET loading** | **LST loading** | **NDBSI loading** |
| --- | --- | --- | --- | --- | --- |
| 2001 | 65.01 | 0.92038 | 0.38868 | -0.03385 | -0.02616 |
| 2002 | 62.69 | 0.91299 | 0.40544 | -0.04507 | -0.00567 |
| 2003 | 65.75 | 0.93537 | 0.35357 | -0.00297 | -0.00780 |
| 2004 | 67.23 | 0.93035 | 0.36367 | -0.04602 | -0.00857 |
| 2005 | 67.90 | 0.93241 | 0.35937 | -0.03709 | -0.00934 |
| 2006 | 61.90 | 0.93143 | 0.36068 | -0.04831 | -0.00494 |
| 2007 | 69.79 | 0.93153 | 0.36364 | -0.00167 | -0.00277 |
| 2008 | 66.17 | 0.94132 | 0.33710 | -0.01694 | -0.00069 |
| 2009 | 65.86 | 0.93482 | 0.35377 | -0.02398 | -0.01974 |
| 2010 | 67.77 | 0.94216 | 0.32093 | -0.07627 | -0.05936 |
| 2011 | 68.34 | 0.94749 | 0.31708 | -0.01123 | -0.04009 |
| 2012 | 64.84 | 0.93257 | 0.33838 | -0.11168 | -0.05779 |
| 2013 | 66.18 | 0.94707 | 0.32094 | -0.00754 | -0.00152 |
| 2014 | 65.79 | 0.94560 | 0.32007 | -0.04784 | -0.03320 |
| 2015 | 68.61 | 0.94346 | 0.33125 | -0.00681 | -0.01022 |
| 2016 | 68.46 | 0.94572 | 0.32347 | -0.00945 | -0.02977 |
| 2017 | 66.87 | 0.92726 | 0.37029 | -0.05423 | -0.01167 |
| 2018 | 70.00 | 0.93386 | 0.34863 | -0.01392 | -0.07862 |
| 2019 | 67.01 | 0.93762 | 0.34714 | -0.01718 | -0.00809 |
| 2020 | 71.27 | 0.91900 | 0.38390 | -0.08900 | -0.01226 |
| 2021 | 73.96 | 0.95353 | 0.29853 | -0.00567 | -0.04043 |
| 2022 | 66.56 | 0.93493 | 0.33914 | -0.10388 | -0.01008 |

Note: PCA was performed independently for each autumn season-year combination (September-November).

**Table S4. Variance inflation factors (VIFs) for explanatory variables used in lagged SPEI partial-correlation models.**

| Target season | SPEI candidate | Lagged SPEI | Radiation | Temperature | Precipitation | Soil moisture | Human footprint |
| --- | --- | --- | --- | --- | --- | --- | --- |
| Spring | Previous summer SPEI-3 | 1.01 | 1.47 | 1.05 | 1.45 | 1.04 | 1.00 |
| Spring | Previous autumn SPEI-3 | 1.02 | 1.47 | 1.07 | 1.45 | 1.04 | 1.00 |
| Spring | Winter SPEI-3 | 1.08 | 1.48 | 1.10 | 1.46 | 1.05 | 1.00 |
| Spring | Spring SPEI-3 | 2.15 | 1.66 | 1.35 | 1.70 | 1.04 | 1.00 |
| Summer | Previous autumn SPEI-3 | 1.00 | 2.04 | 1.30 | 1.90 | 1.08 | 1.00 |
| Summer | Winter SPEI-3 | 1.00 | 2.03 | 1.30 | 1.88 | 1.08 | 1.00 |
| Summer | Spring SPEI-3 | 1.01 | 2.04 | 1.30 | 1.88 | 1.08 | 1.00 |
| Summer | Summer SPEI-3 | 2.28 | 2.36 | 1.35 | 2.12 | 1.10 | 1.00 |
| Autumn | Winter SPEI-3 | 1.02 | 1.70 | 1.08 | 1.74 | 1.10 | 1.01 |
| Autumn | Spring SPEI-3 | 1.01 | 1.71 | 1.07 | 1.75 | 1.10 | 1.01 |
| Autumn | Summer SPEI-3 | 1.03 | 1.70 | 1.07 | 1.74 | 1.12 | 1.01 |
| Autumn | Autumn SPEI-3 | 2.23 | 1.86 | 1.19 | 2.13 | 1.11 | 1.01 |

**Figure S1. Spatial distribution of aridity zones in China based on the mean aridity index during 2001-2022. Aridity zones were classified as humid, semi-humid, semi-arid and arid regions according to the long-term mean AI.**

**References**

Hirsch, R.M., Slack, J.R., and Smith, R.A. (1982). Techniques of trend analysis for monthly water quality data. *Water Resources Research* 18(1)**,** 107-121. doi: 10.1029/WR018i001p00107

Kendall, M.G. (1975). *Rank Correlation Methods.* London: Charles Griffin.

Li, Y., Tian, H., Zhang, J., Lu, S., Xie, Z., Shen, W., et al. (2023). Detection of spatiotemporal changes in ecological quality in the Chinese mainland: Trends and attributes. *Science of The Total Environment* 884**,** 163791. doi: 10.1016/j.scitotenv.2023.163791

Mann, H.B. (1945). Nonparametric tests against trend. *Econometrica* 13**,** 245-259. doi: 10.2307/1907187.

Sen, P.K. (1968). Estimates of the Regression Coefficient Based on Kendall's Tau. *Journal of the American Statistical Association* 63(324)**,** 1379-1389. doi: 10.1080/01621459.1968.10480934

Xu, H. (2013). A remote sensing index for assessment of regional ecological changes. *China Environmental Science* 33(5)**,** 889-897.
